# Different Approaches to Develop Nanosensors for Diagnosis of Diseases

**DOI:** 10.1002/advs.202001476

**Published:** 2020-10-28

**Authors:** Nina Arndt, Huong D. N. Tran, Run Zhang, Zhi Ping Xu, Hang T. Ta

**Affiliations:** ^1^ Queensland Micro‐ and Nanotechnology Centre Griffith University Brisbane Queensland 4111 Australia; ^2^ Australian Institute for Bioengineering and Nanotechnology the University of Queensland Brisbane Queensland 4072 Australia; ^3^ Department of Biotechnology Technische Universität Berlin Berlin 10623 Germany; ^4^ School of Environment and Science Griffith University Brisbane Queensland 4111 Australia

**Keywords:** disease diagnosis, microsensors, nanosensors

## Abstract

The success of clinical treatments is highly dependent on early detection and much research has been conducted to develop fast, efficient, and precise methods for this reason. Conventional methods relying on nonspecific and targeting probes are being outpaced by so‐called nanosensors. Over the last two decades a variety of activatable sensors have been engineered, with a great diversity concerning the operating principle. Therefore, this review delineates the achievements made in the development of nanosensors designed for diagnosis of diseases.

## Graphical abstract

Activatable nanosensors are a promising technology platform for detecting and grading diseases efficiently even at early stage of disease. This literature review aims to delineate the progression of nanosensor designs regarding their principles of activation and application. Nanosensors can be activated via target interaction with enzyme, RNA, DNA, protein, or via physiological changes induced by pH, reactive oxygen species/nitrogen species, or glutathione interaction.

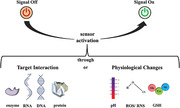

## 1 Introduction

The success of clinical treatment is highly depended on early detection. Detecting tumors before metastasis improves the survival rate,^[^ ^1^, ^2^ ^]^ and identifying vulnerable plaques before rupture may prevent thrombosis and therefore lower the risk of myocardial infarction and stroke.^[^ ^3^ ^]^ Furthermore, early detection preserves the patient's life quality and reduces costs. Many of the current medical imaging methods, such as magnetic resonance imaging (MRI), computed tomography (CT), and near infrared fluorescence molecular tomography (NIR FMT), require imaging probes to produce a signal or contrast enhancement. These imaging probes can be distinguished as nonspecific probes used for imaging physiological processes, targeted probes that specifically bind to their target, and activatable sensors whose signal is altered at the target site or under specific physiological conditions.^[^ ^4^ ^]^

Paramagnetic chelated gadolinium^[^ ^4^, ^5^ ^]^ or superparamagnetic iron oxide nanoparticles (IONP)^[^ ^4^ ^]^ are commonly used as nonspecific or targeted probes in MRI. Recent attempts tried to combine both properties, creating dual contrast agents with a T_1_ and T_2_ enhancing effect.^[^ ^6^ ^]^ Iodinated contrast agents are used in X‐ray and computed tomography (CT) to improve the soft‐tissue contrast.^[^ ^4^ ^]^ However, nonspecific and targeted probes cause a low signal‐to‐noise ratio. The use of targeted probes often requires the physiological clearance of unbound probes, resulting in longer diagnosis time and reducing the contrast because of probe consumption.^[^ ^4^, ^7^ ^]^ Activatable sensor probes improve the signal‐to‐background ratio and are therefore highly advantageous over nonspecific and targeted probes.

Over the last decades, different types of activatable sensors for diagnosis of disease have been designed such as small molecule sensors^[^ ^8^ ^]^ as well as nanosensors.^[^ ^9^, ^10^, ^11^, ^12^, ^13^ ^]^ While small molecule sensors are designed to respond to the presence of small molecules such as ions (e.g., cooper, iron or zinc)^[^ ^14^ ^]^ and reactive oxygen species (ROS)^[^ ^8^ ^]^; nanosensors interact with proteins,^[^ ^9^, ^10^, ^11^, ^12^, ^13^ ^]^ nucleic acids,^[^ ^15^, ^16^, ^17^ ^]^ and also with ions or respond directly to physiological changes.^[^ ^18^, ^19^, ^20^ ^]^ Since alterations concerning the concentration of metal ions or ROS are associated with certain diseases, small‐molecule sensors have been shown to be a promising approach for disease diagnosis. They usually consist of a fluorophore whose fluorescence is altered when the corresponding small molecule binds to the fluorophore. Consequently, the response of these sensors is measured through optical imaging.^[^ ^14^ ^]^ Optical imaging is also a common method for monitoring nanosensor activation.^[^ ^9^, ^10^, ^11^, ^12^ ^]^ Additional imaging methods such as MRI^[^ ^13^ ^]^ as well as electrochemical based detection^[^ ^21^ ^]^ are conventional methods for examining the sensor response. Nanosensors and small molecule sensors offer promising approaches for disease detection. Considering the spectrum of biomarkers and detection methods of these two sensor types, nanosensors offer a higher variety. This review will only discuss nanosensors for diagnosis of disease.

Developing sensors at the nanoscale has several benefits as particles in this size range exhibit special characteristics. Nanoprobes have been shown to offer a high penetration efficiency^[^ ^22^ ^]^ and may be taken up by cells naturally.^[^ ^23^ ^]^ Using nanomaterials as carriers of chemical probes can increase the stability and the half‐life of the chemical probes.^[^ ^22^ ^]^ Due to their small size, nanoparticles show a high surface‐to‐volume ratio, which enables high‐sensitive detection even at femto‐, atto‐, and zepto‐scales.^[^ ^24^ ^]^ Furthermore, nanoparticles are shape‐tunable and can exhibit high electrical conductivity and reactivity.^[^ ^24^ ^]^

**Scheme 1:**
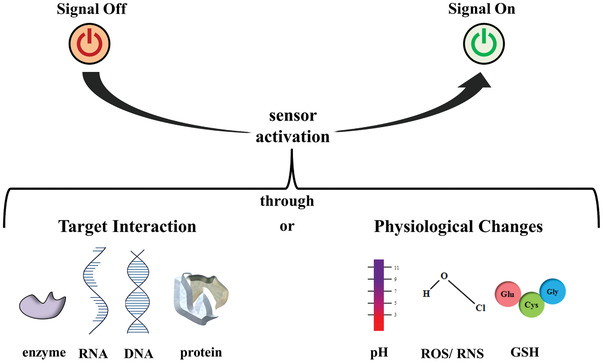
Summary of the different approaches to activate nanosensors via enzyme, RNA, DNA, protein, pH, ROS/RNS, or GSH interaction.

Activatable nanosensors are a promising technology platform for detecting and grading diseases efficiently even at early stage of disease. Having highlighted their potential and importance, this literature review aims to delineate the progression of nanosensor designs regarding their principles of activation and application (**Scheme** **1**).

Reviews on nanosensors already exist, however, these focus on the detection of particular biomarkers,^[^ ^25^, ^26^ ^]^ on the application of a certain material^[^ ^27^, ^28^, ^29^ ^]^ or on the diagnosis of specific diseases such as celiac disease,^[^ ^30^ ^]^ tuberculosis,^[^ ^31^ ^]^ and hepatitis.^[^ ^32^ ^]^ Other reviews address a certain type of nanosensor such as protease nanosensors (from 2010),^[^ ^33^ ^]^ sensors for molecular MRI^[^ ^34^ ^]^ or are specialized on point‐of‐care diagnostics.^[^ ^35^ ^]^ Therefore, in this review we wish to give a comprehensive and global presentation on the development and the variety of nanosensor designs for the diagnosis of diseases.

A variety of activatable sensors designed for diseases diagnosis, from in vivo to in vitro, have been engineered over the last two decades with a great diversity concerning the operating principle. The first part of this review covers nanosensor that are activated in vivo, while the second focuses on in vitro nanosensor activation. Both parts are structured similarly. At first, sensors that are activated via target interaction are presented followed by the depiction of nanosensors activated by physiological changes. The target‐interaction section is large and therefore, substructured into sensor activation through biocatalytic events and through binding events. Furthermore, different detection methodologies (such as optical imaging, magnetic resonance imaging, magnetic particle spectroscopy, enzyme‐linked immunosorbent assay (ELISA), and lateral flow assay) will be presented in conjunction to the nanosensor activation principles.

## 2 In Vivo Nanosensors

As mentioned before, in vivo nanosensors can be grouped according to their activation principles. Here, nanosensors that are activated via target interaction will be discussed first, followed by those activated due to physiological changes.

### 2.1 Activation via Target Interaction

Activation via target interaction is possible due to biocatalytic events, such as protease activity, and binding/hybridization event. Concerning the latter, the binding of a biomolecule causes conformational changes in the nanosensor structure leading to the activation of the sensor.

#### 2.1.1 Biocatalytic‐Dependent Activation

Overexpression of proteases is associated with a variety of diseases such as cancer, neurodegenerative disorders, cardiovascular diseases, and gastric ulcer. In some cases, protease activity can even serve as indicator for the disease stage.^[^ ^11^, ^36^ ^]^ Therefore, targeting proteases and monitoring their activity is a promising method for the diagnosis and the grading of diseases.

The most common tool for monitoring protease activity is optical imaging. Optical imaging relies on signal quenching of dyes in their native state due to their autoquenching in close proximity,^[^ ^9^, ^10^, ^11^, ^37^, ^38^, ^39^, ^40^, ^41^, ^42^, ^43^, ^44^ ^]^ Förster resonance energy transfer (FRET),^[^ ^45^ ^]^ bioluminescence resonance energy transfer (BRET)^[^ ^46^, ^47^ ^]^ or a quencher property in form of gold^[^ ^48^, ^49^ ^]^ or an iron oxide nanoparticle (IONP).^[^ ^40^, ^50^ ^]^ The fluorescent dyes are attached to their quenching moiety over a peptide containing a cleavage site for a particular protease. In the presence of the respective protease, the peptide is cleaved, dissevering the dye, and restoring the signal. The quenching through FRET occurs due to a nonradiative energy transfer between an energy donor and an energy acceptor caused by dipole–dipole interaction. Resonance energy transfer only occurs when the emission spectrum of the energy donor and the absorption spectrum of the energy acceptor overlap, the donor and acceptor are within 1–10 nm proximity and the dipole moments are in the right orientation. When the donor is excited and the energy transferred to the acceptor, the fluorescence of the donor is quenched and the energy may be emitted by the energy acceptor via fluorescence.^[^ ^51^ ^]^ In case of BRET, the energy donor emission is due to bioluminescence.^[^ ^52^ ^]^

##### In‐Body Detection

In vivo nanosensors interact with their target in the body. However, sensor activation can be detected directly in the body or outside of the body (e.g., urinary reporter). The following section will discuss the in‐body detection of in vivo nanosensors.

- Optical Detection

**Figure 1:**
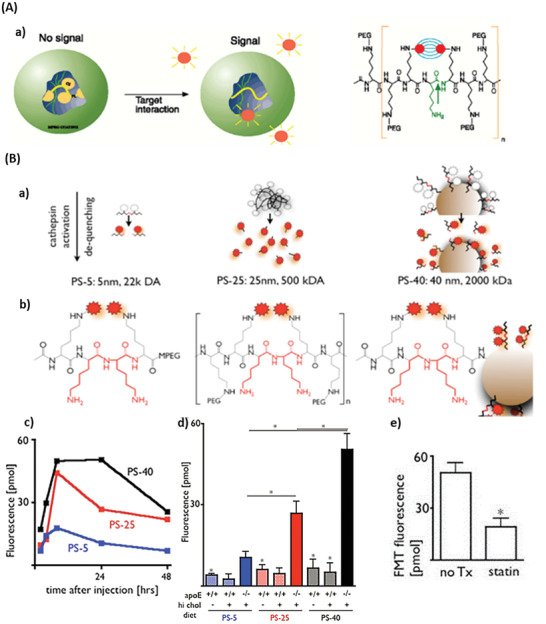
NIRF protease activatable probe. A) Operating principle and design of NIRF sensors for monitoring protease activity. a) The sensor is inactive in its native state. Due to the close proximity of the fluorescent dyes the signal is quenched. In presence of the respective protease the substrate is cleaved, thus, separating the dyes and restoring the fluorescence. b) Chemical structure of the graft copolymer. The green arrow shows the position of protease cleavage. Reproduced with permission.^[^ ^9^ ^]^ Copyright 1999, Springer Nature. B) Operating principle, chemical structure, and experimental results of PS5, PS25, and PS40. a) Schemata of PS5, PS25, and PS40. The white jagged circles indicate quenched dyes, while the red ones represent active fluorescent dyes. b) Chemical structure of PS with protease cleavage site highlighted in red. c) In vivo FMT imaging of the fluorescence over time in apoE^−/−^ mice. d) In vivo FMT imaging of the fluorescence 24 h after the sensors were injected in apoE^−/−^ and wild type mice on normal and on high fat diet. e) FMT imaging of apoE^−/−^ mice treated with atorvastatin 24 h after PS40 was injected. Reproduced with permission.^[^ ^40^ ^]^ Copyright 2009, Wolters Kluwer Health.

*Optical Imaging Based on Autoquenching*: Weissleder et al.^[^ ^9^ ^]^ were one of the earliest scientists to develop a biocompatible protease‐activated near infrared fluorescence (NIRF) probe that generated a strong NIRF signal to detect micrometer‐sized tumors. They used a novel, long‐circulating, nontoxic clinically available graft‐copolymer containing cleavage sites for lysin–lysin active proteases (e.g., trypsin and cathepsin B) that are known to accumulate naturally in tumors. An average of 91 methoxypolyethylene glycols (MPEG) along the backbone of the graft polymer ensured sterical protection, and the attachment of NIRF dyes (Cy5.5) enabled optical detection of targeting protease (**Figure** **1**A).^[^ ^9^ ^]^ The use of near infrared (NIR) reporters such as Cy5.5 is highly advantageous over visible or infrared light probes, because tissues show low absorbance in the near‐infrared spectrum but a high scattering capacity.^[^ ^53^ ^]^ In close proximity, the fluorescent signal of the NIR dye is autoquenched due to energy resonance transfer. In the presence of proteases with lysin–lysin cleavage activity, the polymer is degraded, separating the reporter probes from each other and restoring the signal. Weissleder et al.^[^ ^9^ ^]^ performed experiments, suggesting that the NIRF probe was taken up by a variety of different tumors (including LX‐1 tumor cells, C6 glioma, 9L gliosarcoma, R13762 adenocarcinoma, LX‐1 small cell lung carcinoma, MCF‐7 mammary adenocarcinoma, and colon adenocarcinoma) and activated by serine (trypsin) and cysteine (cathepsin B, H, and L) proteases,^[^ ^9^ ^]^ which play an essential role in tumor invasion, angiogenesis, and metastasis.^[^ ^54^, ^55^, ^56^ ^]^ The cathepsin‐sensitive NIRF probes were able to detect human breast cancer carcinoma (BT‐20) with the size as small as 1 mm in nude mice^[^ ^38^ ^]^ and adenomatous polyps as small as 50 µm in diameter.^[^ ^44^ ^]^ Further successful imaging and quantification experiments using the same novel NIRF probe were demonstrated on 9L gliosarcoma and HT1080 human fibrosarcoma cells implanted into nude mice.^[^ ^37^ ^]^

Cathepsin B is a tumor‐active protease, but also overexpressed and secreted by activated macrophages and therefore associated with plaque erosion and rupture.^[^ ^40^, ^57^ ^]^ Chen et al. applied the cathepsin B‐sensitive NIRF probe (with the same structure as just described^[^ ^9^ ^]^) in murine model to determine the potential of atherosclerosis associated activation of the cathepsin B‐sensitive probe using fluorescence‐mediated tomography (FMT). A strong fluorescence signal was detected in atherosclerotic lesions, indicating the potential of cathepsin B‐sensitive NIRF probes to measure plaque inflammation and vulnerability.^[^ ^10^ ^]^ This protease sensing probe is advantageous over other imaging techniques measuring stenosis, since luminal narrowing is a poor indicator for plaque vulnerability.^[^ ^10^ ^]^

Tung et al. introduced a similar cathepsin D activatable probe. Fluorescein isothiocyanate (FITC)‐labeled peptides with cathepsin D specificity were conjugated to a protected graft copolymer (PGC) (as noted before, consisting of MPEG sidechains along a poly‐_L_‐lysine backbone), making this probe sensitive for cathepsin D instead of cathepsin B. The peptide has the sequence *Gly‐Pro‐Ile‐Cys(Et)‐Phe‐Phe‐Arg‐Leu‐*Gly‐Lys(FITC)‐Cys‐NH_2_, whereas the italic letters indicate the Cathepsin D substrate.^[^ ^42^ ^]^ Cathepsin D is an aspartic proteinase and its overexpression is associated with metastasis, tumor invasion, angiogenesis and degradation of the basement membrane, and therefore cathepsin D serves as adequate indicator of tumor progression. Tung et al. demonstrated for the first time that cathepsin D activity can be imaged in vivo through NIRF imaging sensors by using cathepsin+ and cathepsin− rodent tumors implanted into nude mice.^[^ ^41^ ^]^

The NIRF sensor structure proposed by Tung et al. offers a high tunability concerning the peptide protease specificity. Consequently, this nanosensor can be used for detection of different proteases. Bremer et al.^[^ ^11^ ^]^ used a gelatinase matrix metalloproteinase‐2 (MMP‐2) peptide substrate (Gly‐*Pro‐Leu‐Gly‐Val‐Arg‐*Gly‐Lys(FITC)‐Cys‐NH_2_, whereas the italic letters indicate the MMP‐2 substrate) instead of a cathepsin D specific peptide to sense MMP activity in tumors. Extracellular matrix remodeling proteinase such as MMPs have been known for playing a pivotal role in carcinogenesis by being involved in tumor growth, invasion, metastasis, and angiogenesis. Hence, MMPs are valuable diagnostic and therapeutic targets for the detection, grading and treatment of cancer.^[^ ^58^, ^59^ ^]^ Bremer et al. developed an NIRF probe which was able to monitor different MMP‐2 activities in HT1080 and BT20 tumor bearing mice and record the efficiency of protease inhibitors such as phenanthroline.^[^ ^11^ ^]^

Nahrendorf et al. introduced three cysteine protease sensors (PS) for in vivo FMT imaging of atherosclerotic plaques,^[^ ^40^ ^]^ i.e., PS5, PS25, and PS40 (5, 25, and 40 nm sized protease‐based nanosensors, respectively). All three protease sensors were based on a fluorophore (VivoTag‐S680) labeled oligo‐l‐lysine peptide containing a cleavage site for cathepsin B and varied in size and design. PS5 and PS25 sized protease sensors were made up of a fluorochrome labelled peptides (FLP), while for PS40 FLPs were conjugated to amine‐functionalized polymeric nanoparticles (NP) (Figure 1B). The fluorescent signal was autoquenched due to the close proximity of the NIR dyes. The iron oxide nanoparticles (IONP) enhanced the quenching effect for PS40. Their experiments showed that the fluorescence intensity for all three probes was similar in vitro, but PS40 quenched the signal most efficiently and therefore showed the highest increase in fluorescence. Furthermore, PS40 showed the lowest wash‐out kinetics (Figure 1B). Ex vivo and in vivo experiments demonstrated that activation of all three sensors was higher in mice with atherosclerosis than in wild‐type mice. The highest fluorescence was achieved for PS40 (Figure 1B), indicating that the use of an iron oxide core as a “cellular anchor” enhances sensitivity. Therefore, Nahrendof et al. investigated the potential of PS40 to monitor the success of statin therapy in vivo by treating apolipoprotein E knockout (ApoE^−/−^) mice with atorvastatin, which reduced the recruitment of cathepsin expressing inflammatory monocytes, thus, decreasing the activation of PS40. ApoE^−/−^ mice are prone to develop atherosclerosis,^[^ ^60^ ^]^ however, when treated with atorvastatin, the PS40 fluorescent molecular tomography (FMT) signal in the aortic root was reduced by 2.6‐fold (Figure 1B), indicating the potential of this sensor for tracking drug effects in mice.^[^ ^40^ ^]^

**Figure 2:**
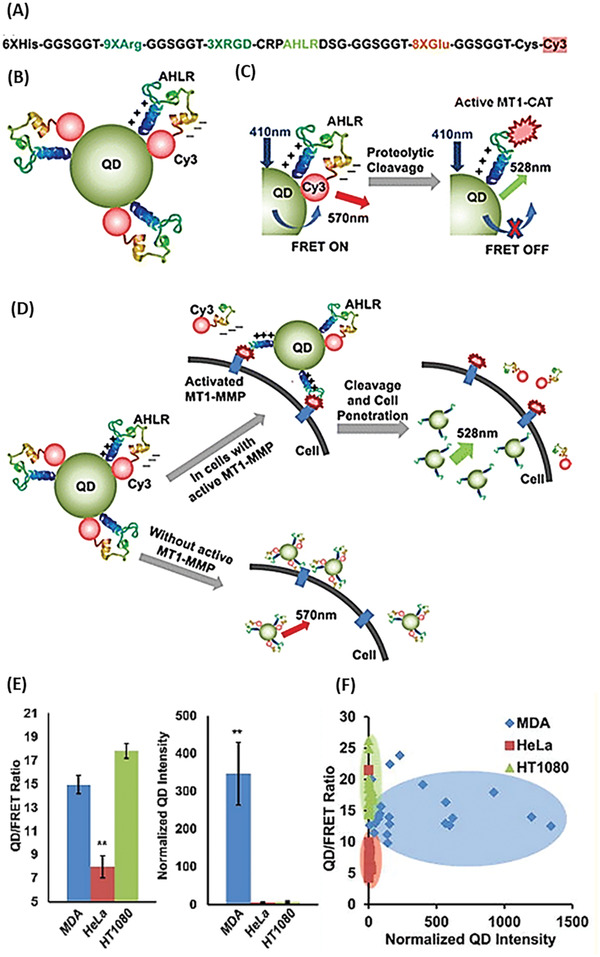
Operating principle and experimental data of the QD/FRET nanosensor. A) Sequence of the MT1‐MMP cleavable peptide. B) Cy3 is linked to the QD via a MT1‐MMP cleavable peptide, enabling FRET. C) The sensor becomes activated by cleavage of the peptide through MT1‐MMP, disabling FRET. D) In presence of the respective protease, the peptide is cleaved, and FRET donor and acceptor are separated. This can trigger the cellular uptake. E) QD/FRET ratio (left diagram) and QD intensity (right diagram) in MDA‐MB‐231, Hela and HT 1080 cells. F) QD/FRET ratio plotted against normalized QD intensity in MDA‐MB‐231, Hela and HT 108 cells. Reproduced with permission.^[^ ^45^ ^]^ Copyright 2015, American Chemical Society.

*Optical Imaging Based on FRET*: Semiconductor quantum dots (QD) have been shown to be efficient energy donors in FRET for biological sensing. A variety of protease sensing QD/FRET sensors have emerged over the last decade.^[^ ^45^, ^61^, ^62^, ^63^, ^64^, ^65^, ^66^, ^67^, ^68^, ^69^, ^70^ ^]^ For instance, Chung et al.^[^ ^45^ ^]^ developed a QD/FRET protease nanosensor for imaging membrane‐type‐1 matrix metalloproteinase (MT1‐MMP) and integrin receptor expression in single cells with the aim to profile cancer cell potential of tissue invasiveness and degradation. The QD/FRET protease nanosensor consisted of a Cd/ZnS QD coupled to multiple Cy3 bendable peptides (6xHis‐Gly‐Gly‐Ser‐Gly‐Gly‐Thr‐9x(Arg)‐Gly‐Gly‐Ser‐Gly‐Gly‐Thr‐3x(Arg‐Gly‐Asp)‐Cys‐Arg‐Pro‐*Ala‐His‐Leu‐Arg‐*Asp‐Ser‐Gly‐Gly‐Gly‐Ser‐Gly‐Gly‐Thr‐8x(Glu)‐Gly‐Gly‐Ser‐Gly‐Gly‐Thr‐Cys‐Cy3) via the hexa‐histidine sequence of the bendable peptide (**Figure** **2**A). The peptide contained a cleavage site for MT1‐MMP (Ala‐His‐Leu‐Arg, indicated in italic in the previous sequence) and a cell targeting site (3x (Arg‐Gly‐Asp)). Electrostatic attractions between nine cationic arginines and eight anionic glutamates ensured a hairpin structure and enabled FRET between QD as the FRET donor and Cy3 as the FRET acceptor (Figure 2B). The bending of the peptide was supported by the flexible linker sequences (Gly‐Gly‐Ser‐Gly‐Gly) flanking the arginine and glutamate sequence. The cell target site of the sensor attached to the cell expressed surface receptor integrin. In presence of MT1‐MMP the peptide was cleaved, separating the FRET donor and acceptor, therefore, changing the emission (from 570 nm in its native state to 528 nm when excited with 410 nm light (Figure 2C). The 9xArg sequence that is exposed after MT1‐MMP cleavage served as cell‐penetrating peptide, allowing the entering of the nanosensor into the cell (Figure 2D). By evaluating the QD/FRET ratio and the normalized QD intensity, the sensor was shown to reflect the MT1‐MMP activity and the cellular uptake of QDs in different cell lines, namely, MDA, Hela and HT 1080 and to allow cell‐line differentiation. Since MT1‐MMP expression is high in MDA and HT 1080 cell lines, sensors became activated, causing a greater QD/FRET ratio compared to Hela cells. By monitoring the normalized QD intensity, it was possible to draw conclusions concerning the cellular uptake. Consequently, MDA cells showed a high QD intensity while the one of Hela and HT 1080 cells was low (Figure 2E,F).^[^ ^45^ ^]^

Another tumor detecting QD/FRET nanosensor targeting MMP‐2 was designed by Li et al.^[^ ^70^ ^]^ consisting of a CdTeS QD linked to the NIR dye ICG‐Der‐02 over a MMP‐2 cleavable peptide (Gly‐Pro‐Leu‐Gly‐Val‐Arg‐Gly‐Lys‐Gly‐Gly).^[^ ^70^ ^]^ QD/FRET nanosensors for monitoring caspase‐1,^[^ ^61^, ^64^, ^66^ ^]^ chymotrypsin, thrombin and collagenase activity were engineered by Medintz et al.^[^ ^61^ ^]^

Other groups developed human immunodeficiency virus protease (HIV‐1 PR) (a protease essential for the infectivity of the viral particles) sensing probes for recording protease activity in transfected cells and protease inhibitor effectiveness. The latter aimed to address the problem of emerging drug resistant viral variations.^[^ ^65^, ^67^ ^]^ Again, these sensors consisted of the FRET acceptors linked to a QD as energy donor via modular peptides containing a cleavage site for the respective protease. Here, for HIV‐1PR, the peptide of Ser‐Gln‐Asn‐Tyr‐Pro‐Ile‐Val‐Gln was employed.^[^ ^65^, ^67^ ^]^ The overall structure of these sensors is the same.

A common nonfluorogenic quencher used to pair with organic fluorochrome or semiconductors with fluorescence in the visible light range (400–650 nm) is 4‐(4′‐dimethylaminobenzeneazo) benzoic acid (DABCYL)^[^ ^71^ ^]^ as used in one of the described HIV‐1PR monitoring nanosensors.^[^ ^65^ ^]^ As mentioned previously, it is highly recommended and advantageous to use fluorescent probes in the near infrared range (NIR), so NIR probes require a quencher analogous to DABCYL in the NIR absorption spectrum. Palm et al. proposed an azulene NIR quencher (NIRQ) probe coupled over a caspase‐3 substrate (Gly‐Asp‐Glu‐Val‐Asp‐Gly‐Ser‐Gly‐Cys) to Alexa‐680. When incubating the probe with the targeting enzyme, a four‐fold fluorescent increase was detected.^[^ ^71^ ^]^ In a later study the group reported a better efficiency for the NIR absorber NIRQ820. The NIRFQ820 was coupled over an MMP‐7 substrate (Gly‐Val‐Pro‐Leu‐Ser‐Leu‐Thr‐Met‐Gly‐Cys) to Cy5.5 to image tumor‐associated protease activity.^[^ ^72^ ^]^

**Figure 3:**
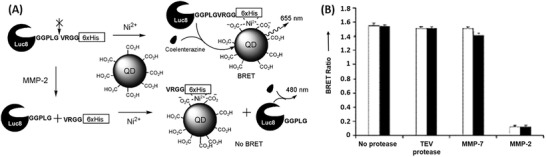
Operating principle and experimental data of BRET‐based detection of MMP‐2. A) BRET‐based detection of MMP‐2 with assembled QD nanosensors. In presence of MMP‐2, Luc8 is separated from the QD preventing BRET. B) BRET ratio of the QD/BRET nanosensor when Luc8‐peptide conjugate was pretreated with no protease, tobacco etch virus (TEV) protease, MMP‐7 or MMP‐2 for 2 h (white column) or 6 h (black column). Reproduced with permission.^[^ ^46^ ^]^ Copyright 2007, Wiley‐VCH.

*Optical Imaging Based on BRET*: In the previous example, QDs serve as an energy donor in FRET.^[^ ^45^ ^]^ In fact, QDs can also serve as an energy acceptor from a bioluminescent fusion protein in bioluminescent resonance energy transfer (BRET). Self‐illuminating QDs do not require any external excitation and therefore offer a high sensitivity with low background emission. BRET also shows a greater spectral separation of donor and acceptor emission compared to FRET.^[^ ^46^, ^47^ ^]^ BRET/QDs have been shown to be compatible with both bioluminescence and fluorescence imaging.^[^ ^73^ ^]^ So et al. proposed self‐illuminating QDs using R. reniformis luciferase (Luc8) as light emitting protein. Luc8 was conjugated to CdSe/ZnS core–shell QDs (QD655‐Luc8) via an amide‐bond between the amino groups at the surface of Luc8 and the carboxylates of the QD. They gave an emission peak at 480 nm upon addition of its substrate, coelenterazine. QD655‐Luc8 nanosensor emitted a new peak at 655 nm due to the occurrence of BRET. The released energy from oxidation of the substrate is transferred from the luciferase to the QDs through BRET.^[^ ^73^, ^74^ ^]^ They demonstrated that BRET emission was imaged in cells and small animals, and the potential of the proposed QD655‐Luc8 probe to label cells, and monitor these cells in vivo.^[^ ^73^ ^]^ Wu et al. used this probe for in vivo mapping and imaging of sentinel lymph node (SLN) in nude mice. The study showed that self‐illuminating QD‐bioluminescence structure greatly enhanced the sensitivity compared to standard fluorescent QDs.^[^ ^47^ ^]^ Yao et al. also developed a QD/BRET nanosensor for recording the activity of MMP‐2. The construct was similar to the QD655‐Luc8 nanosensor with a peptide containing a MMP substrate (Gly‐Gly‐Pro‐Leu‐Gly‐Val‐Arg‐Gly‐Gly‐6x(His)) and six histidine tags, which formed complexes with the carboxylic acids on the QD in presence of Ni^2+^ cations. In the presence of MMP, the luciferase was separated from the QD, preventing the occurrence of BRET. In the absence of the tumor protease, the nanosensor stayed intact and a QD signal was detected when adding the luciferase substrate, coelenterazine (**Figure** **3**A). Proteases other than MMP‐2 showed no significant change of the BRET ratio indicating the high MMP‐2 protease sensitivity of this sensor (Figure 3B). By exchanging the substrate this sensor can be used for detecting other hydrolytic enzymes and its application can be extended to multiplexed detection of bioanalytes.^[^ ^46^ ^]^

**Figure 4:**
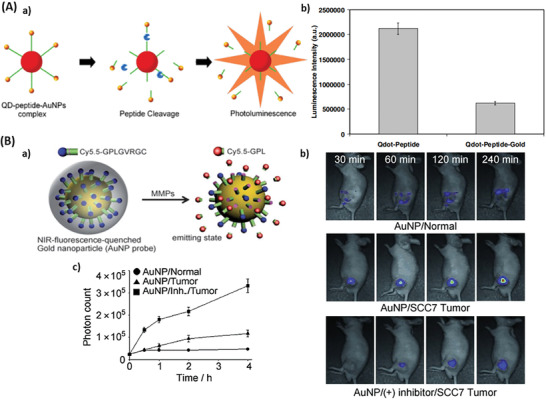
Probes employing quenching property of AuNPs. A) QD‐AuNP sensor for collagenase activity. a) Activation of QD/AuNP sensor. In presence of the respective protease, the peptide linking QD and AuNP is cleaved and the QD signal restored. b) Luminescence intensity of the QD‐Peptide conjugate (here named Qdot‐Peptide) and of the full nanosensor in its native state (Qdot‐Peptide‐Gold). Reproduced with permission.^[^ ^77^ ^]^ Copyright 2005, Elsevier. B) MMP‐2 activatable AuNP‐Cy5.5 probe. a) Activation of the MMP‐sensing Cy5.5/AuNP sensor. In close proximity of the dyes to AuNP, the fluorescence of Cy5.5 is quenched (blue). In presence of MMP‐2, the substrate is cleaved, and the signal recovered (red). b) NIRF tomography of nude mice without and with SCC7 tumors 30, 60, 120, and 240 min after sensor injection. The last row shows mice that were treated with MMP‐2 inhibitor 30 min before the sensors were injected (blue: low‐intensity, red: high intensity). c) Trend of photon count in the tumor over time. Reproduced with permission.^[^ ^48^ ^]^ Copyright 2008, Wiley‐VCH.

Optical Imaging Based on Quencher Property of Gold or IONP: The previously described probes rely on auto‐quenching, FRET or BRET, and offer sensitive detection of proteases. However, a better quenching efficiency is accomplished through attaching the fluorescent label to gold nanoparticles (AuNPs). This is because of the electronic interaction between the fluorescent dye and the metal nanoparticle, known as surface energy transfer (SET), which is a dipole‐depended nonradiative energy transfer.^[^ ^48^ ^]^ The interaction of an excited fluorescent molecule with metal is highly dependent on the distance between the two as well as on the electronic properties of the metal.^[^ ^75^ ^]^ SET in combination with the self‐quenching effect of the loaded dyes due to static quenching and FRET mechanism improves the quenching efficiency significantly^[^ ^48^ ^]^ and offers a longer valid quenching distance.^[^ ^49^ ^]^ Chang et al. proposed a protease‐activated QD‐AuNP probe for detecting collagenase activity. Collagenases are part of metalloproteinase family and associated with tumor dependent invasion of the extracellular matrix.^[^ ^76^ ^]^ AuNPs were tethered to QDs over a collagenase degradable specific peptide (Gly‐Gly‐Leu‐Gly‐Pro‐Ala‐Gly‐Gly‐Cys‐Gly), whereas the N‐terminus of the peptide was covalently attached to the QD forming an amide bond, quenching the QD photoluminescence efficiently until the peptide was cleaved (**Figure** **4**A). The peptide sequence is adjustable, enabling a great range of applications. To ensure efficient quenching of the QD luminescence through energy transfer the peptide sequence is not allowed to exceed the energy transfer distance. In its native state the QD luminescence of the sensor was quenched with an efficiency of 71% (Figure 4A), indicating the probe's potential for biological applications.^[^ ^77^ ^]^

Lee et al. proposed a NIRF‐quenched AuNP imaging probe for in vivo drug screening and detection of protease activity using NIRF dyes (Cy5.5) loaded on 20 nm AuNP via the MMP substrate peptide (Gly‐*Pro‐Leu‐Gly‐Val‐Arg‐Gly*‐Cys(amide), italic letters indicate the MMP substrate), whereas the N‐terminus was coupled to Cy5.5. The probe is inactive in its native state but activated through MMP‐2 cleavage (Figure 4B). In vitro, in vivo and ex vivo experiments revealed that these probes allowed the monitoring of the activities of both proteases and their inhibitors (Figure 4B). Similar to the previous described systems, this peptide substrate spacer is exchangeable, allowing the recording of different proteases. However intravenously injected AuNPs were not able to clearly detect tumors and were cleared from the bloodstream.^[^ ^48^ ^]^ To overcome the labile surface chemistry of thiol‐gold links in blood, Xie et al. replaced the AuNP with a flower‐shaped Au–Fe_3_O_4_ composite nanoparticle. Three iron oxide nanoparticle (IONP) petals were conjugated to a passivated (or rather thiolpoly(ethylene glycol) modified) AuNP core for stabilization. A bridging substrate with MMP‐2, ‐9, and ‐13 selectivity (Gly‐Pro‐Leu‐Gly‐Val‐Arg‐Gly) coupled to the NIRF dye Cy5.5 was covalently linked to the IONP over tri‐dihydroxyphenylalanine as anchoring unit. The construct is hereafter referred to as flower‐like‐activatable nanoparticles (FANPs). In vitro tests verified the protease‐dependent activation of FANPs, and the improved stability of the composite structure compared to pure gold structures. Besides offering a greater stability, the flower‐like structure also allowed the attachment of more fluorophores, where every fluorophore was quadruply quenched by 1) the close proximity of the dyes themselves 2) the underlying and 3) nearby IONPs, and 4) the AuNP core.^[^ ^49^ ^]^

Mu et al. proposed a nanosensor similar to the one developed by Lee et al. but incorporated another quenching property to improve the quenching effect. Efficient fluorescence quenching is only ensured when fluorophore and AuNP are within a certain proximity, otherwise the AuNP might enhance the fluorescence signal. Therefore, Mu et al. attached Quasar‐670‐labeled peptide substrates to the 20 nm passivated AuNP, and incorporated BHQ2 (Black hole quencher 2)‐labeled peptide substrates to provide additional background fluorescent suppression of Quasar‐670. In its native state, the fluorescent signal was quenched, but the signal was restored upon addition of trypsin (substrate: His‐Ser‐Ser‐Lys‐Leu‐Gln) or urokinase‐type plasminogen (substrate: Gly‐Gly‐Ser‐Gly‐Arg‐Ser‐Ala‐Asn‐Ala‐Lys), which is associated with prostate and breast cancer. The probe was produced in a one‐step reaction, simplifying the synthesis process.^[^ ^78^ ^]^

As previously discussed, Weissleder et al. developed an NIRF cathepsin B‐sensitive probe.^[^ ^9^, ^11^, ^41^ ^]^ To improve the spatial/anatomic signal of this probe, they further developed a combined MR/optical imaging probe by attaching a Cy5.5 labeled peptide (Arg‐Arg‐Arg‐Arg‐Gly‐Cys) via either a thioether or a disulfide linkage to a superparamagnetic nanoparticle (Cy5.5‐CLIO). The IONP quenched the fluorescent signal of Cy5.5. Upon addition of DTT (1,4‐dithiothreitol), the peptide disulfide‐linked to nanoparticles was cleaved and a NIRF signal was detectable. The addition of trypsin activated the thioether linked probe. IONP caused a darkening effect on T_2_‐weighted images and enhanced the MRI contrast. Hence this MR/optical imaging nanosensor provided information about the probe location and its molecular environment.^[^ ^79^ ^]^ The probe also offered a new approach to visualize and accurately resect tumors because Cy5.5‐CLIO could be detected preoperatively through MRI and intraoperatively through optical imaging.^[^ ^80^ ^]^

However, quantification of protease concentration was not possible with theses nanosensors because the absolute value of fluorescence depended on intensity of incident light, depth and size of lesion.^[^ ^33^, ^79^ ^]^ To overcome this problem a dual fluorochrome probe was proposed by Weissleder et al. This nanosensor was featured with one NIRF fluorochrome (Cy5.5) activated by proteases (substrate: Arg‐Arg‐Arg‐Arg‐Gly‐Cys) and another fluorochrome (Cy7) directly attached to amino‐CLIO and protease resistant, so the second one served as the internal standard. The probe offered an improvement of enzyme quantification, because the fluorescence ratio is not affected by lesion and depth.^[^ ^33^, ^81^ ^]^

b. Magnetic Resonance Imaging and Magnetic Particle Spectroscopy Based Detection

Protease‐activated nanosensors can also be detected via magnetic resonance imaging (MRI) and magnetic particle spectroscopy (MPS). MRI is based on the alignment of unpaired nuclear spins when placed in a magnetic field. By applying a radiofrequency impulse for excitement, the alignment of the spins is changed, and the time needed to return to the baseline can be recorded. By altering the excitation impulse and the recording time different magnetic contrasts are achieved. The most recent timing parameters used are the longitudinal (*T*_1_) or transverse relaxation time (*T*_2_). Contrast agents shorten either longitudinal or transverse proton relaxation time and therefore enhance the image contrast. For instance, the aggregation of IONPs greatly shortens *T*_2_.^[^ ^4^, ^24^ ^]^

**Figure 5:**
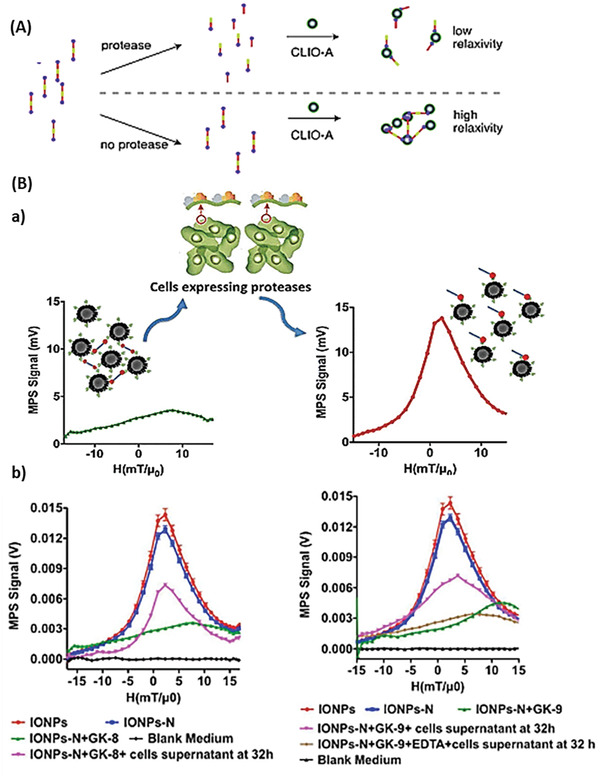
MRI nanosensor based on aggregation of IONP. A) Operating principle of the BBP‐magnetic resonance switcher assay. In the absence of the respective protease, the BBP‐MRS are not cleaved, causing a high relaxivity. The relaxivity is reduced in the presence of the respective protease due to cleavage of the substrate, and thus, no aggregation can occur. Reproduced with permission.^[^ ^12^ ^]^ Copyright 2002, Wiley‐VCH. B) Protease sensing via magnetic particle spectroscopy. a) Operating principle. The sensors are aggregated in the absence of the respective protease. In the presence of the respective protease, the peptide is cleaved and the IONPs dissociate leading to an increased intensity, a decreased full width at half‐maximum and a changed position of the MPS signal. b) MPS signal of dispersed IONPs, neutravidin functionalized IONPs (IONPs‐N), aggregated trypsin sensors (IONPs‐N+GK‐8), dispersed trypsin sensors due to protease cleavage (IONPs‐N+GK‐8+ cells supernatant at 32 h), aggregated MMP‐2 sensors (IONPs‐N+GK‐9), dispersed MMP‐2 sensors due to protease cleavage (IONPs‐N+GK‐9+ cells supernatant at 32 h), aggregated MMP‐2 sensors due to the addition of MMP‐2 inhibitor EDTA (IONPs‐N+GK‐9+EDTA+cells supernatant at 32 h). Reproduced with permission.^[^ ^36^ ^]^ Copyright 2016, American Chemical Society.

Zhao et al. developed a protease assay system to monitor protease activity from the spin–spin relaxation time (*T*_2_) of water molecules. Avidin‐functionalized magnetic nanoparticles (CLIO‐A) (composed of superparamagnetic iron oxide colloid^[^ ^82^ ^]^) appeared in a clustered structure because of the interaction of CLIO‐A with bi‐biotinylated peptide (BBP) substrates. The clustered state induced a high *T*_2_ relaxivity, which was reduced in the presence of proteases due to the cleavage of the substrate sequence between two biotins (**Figure** **5**A, termed as BBP‐magnetic resonance switcher: BBP‐MRS). The study revealed that BBP‐MRS were able to quantify renin (BBP substrate: Arg‐Lys‐(Biotin)‐Ile‐His‐Pro‐Phe‐His‐Leu‐Val‐Ile‐His‐Thr‐Lys‐(Biotin)‐Arg), a protease playing a significant role in regulating blood pressure, in buffer solution containing 2% whole blood. In contrast, a parallel FRET assay showed that the fluorescence of the FRET substrates was reduced near to the background level. Similarly, BBP‐MRS also recorded MMP‐2 activity (BBP substrate: Biotin‐Gly‐Gly‐Pro‐Leu‐Gly‐Val‐Arg‐Gly‐Lys‐(Biotin)) in unpurified cell culture supernatant from HT1080 fibrosarcoma cells. Overall Zhao et al. demonstrated the detection of trypsin, MMP‐2 and renin protease activity using BBP‐MRS.^[^ ^12^ ^]^

A similar principle to BBP‐MRS was used by Gandhi et al. who developed an IONP‐based protease assay using magnetic particle spectroscopy (MPS). In MPS, an altering magnetic field is applied to IONPs in solution, causing a derivation of magnetization which is specifically dependent on the IONP appearance (size, interparticle interactions, surface functionalization, and surrounding environment play a significant role). The engineered nanosensors were based on 22‐nm poly(maleic anhydride‐alt‐1‐octadecene)‐coated IONP functionalized with neutravidin by conjugating the neutravidin amino group to the carboxyl group of the coat via carbodiimide chemistry. The neutravidin functionalized IONPs aggregated through addition of neutravidin cross‐linking molecules, containing a recognition site for either MMP‐2 (cross‐linking molecule: Biotin‐Gly‐Gly‐Pro‐Leu‐Gly‐Val‐Arg‐Gly‐Lys‐Biotin) or trypsin (cross‐linking molecule: Biotin‐ Gly‐Pro‐Ala‐Arg‐Leu‐Ala‐Ile‐Lys‐Biotin). In the presence of MMP‐2 or trypsin, the linker peptide was cleaved and the particles redispersed, causing a different MPS signal (Figure 5B). The application of EDTA as an MMP inhibitor prevented the cleavage of the peptide, leaving the sensors in an aggregated mode. Gandhi et al. anticipated that the proposed nanosensor could be used to detect proteases quantitatively in biological environments, such as tumor microenvironments.^[^ ^36^ ^]^

A similar yet converse approach relying on MRI was presented by Harris et al. Rather than using protease activity to prevent the assembly of nanoparticles, this system facilitated the clustering of the particles. IONPs (50 nm) modified with either neutravidin or biotin were further functionalized with linear polyethylene glycol (PEG) chains via an MMP‐2 peptide (Gly‐Pro‐Leu‐Gly‐Val‐Arg‐Gly‐Cys). The MMP‐2 peptide contained the MMP‐2 substrate sequence, a lysin for linking it to PEG and a cysteine for the attachment onto the neutravidin or the IONP. Linear PEGs of appropriate length (≥10 kDA) inhibited the IONP clustering. However in presence of MMP‐2, the substrate was cleaved, releasing the PEG and allowing the nanosensor self‐assembly, therefore dephasing the surrounding water molecules and causing a shortening of *T*_2_ relaxation time.^[^ ^83^ ^]^

IONPs have been developed as effective imaging contrast agents for molecular MRI of cardiovascular diseases.^[^ ^13^, ^84^, ^85^, ^86^, ^87^, ^88^, ^89^, ^90^, ^91^, ^92^, ^93^, ^94^, ^95^, ^96^, ^97^, ^98^ ^]^ Recently, Ta et al. developed an activatable magnetic resonance nanosensor based on iron oxide and chelated gadolinium for detecting and discriminating thrombosis.^[^ ^13^ ^]^ As knowing whether a thrombus in a blood vessel is new (fresh) or old (constituted) is very important for physicians to decide a treatment protocol, the nanosensor was designed to be able to switch between *T*_1_‐weighted and *T*_2_‐weighted MRI signal depending on thrombus age or the presence/absence of thrombin (a serine protease) at the thrombus site. This design is based on the following fact. With the direct contact between gadolinium and iron oxide (*T*_1_ and *T*_2_ agents, respectively), the magnetic field generated by a superparamagnetic iron oxide material perturbs the relaxation process of the paramagnetic gadolinium and results in the quenching of the *T*_1_ signal. As the nanosensor bound to an active forming thrombus, thrombin (only active on fresh thrombus) would cleave the li0nkers (Lys‐Lys‐Leu‐Val‐Pro‐Arg‐Gly‐Ser) between gadolinium and the iron oxide core, thus restoring *T*_1_ signal for detecting the fresh thrombus. For aged thrombus, *T*_2_ signal would be detected because the nanosensor was intact. However, the efficiency of this nanosensor has not been validated in vivo yet.

##### Outside of Body Detection

**Figure 6:**
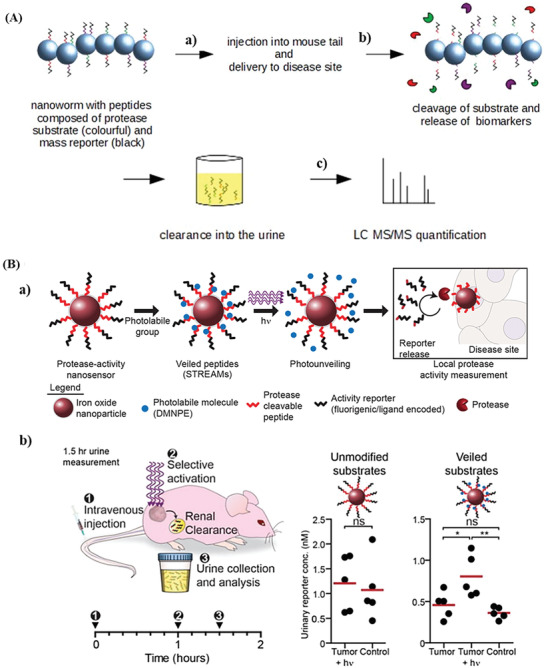
Iron oxide‐based nanoprobes. A) Synthetic urinary biomarker detection. a) The sensors consisted of biomarkers that were conjugated onto a nanoworm. The biomarkers contained a protease cleavage site and a mass‐reporter. b) After intravenous injection, the sensors accumulated at the disease site. The target protease cleaved the respective peptide and the reporters cleared into the urine. c) The quantity of reporters in the urine was analyzed by liquid chromatography tandem mass spectrometry. Redrawn based on^[^ ^100^ ^]^ B) Photoactivatable sensors of protease activity. a) The sensor is sensitive to protease activity as long as the cleavage side is not veiled by a photolabile group. When exposing the sensor to 365 nm light, the photolabile group dissociates and the cleavage site becomes accessible to the respective protease. b) Sensors were injected intravenously. One hour after injection the urine was collected, and the sensors were treated with light for 30 seconds. Results showed that unmodified or unprotected sensors did not distinguish between tumor and healthy mice, while protected or veiled substrates showed a 2.1‐fold increase in tumor mice when treated with light. A 2.6‐fold increase was observed when comparing the signal of veiled substrates treated with light in tumor mice with veiled substrates without light treatment in healthy mice. Reproduced with permission.^[^ ^50^ ^]^ Copyright 2015, American Chemical Society.

While the previous described nanosensors are being detected in vivo, there are more recent attempts trying to design nanosensors that release their reporter element, a synthetic biomarker, into the urine after the enzymatic degradation of the nanosensor by the target disease protease. These probes are based on synthetic biomarkers conjugated to an iron oxide nanoworm via linking peptides containing a cleavage site for a specific protease. The nanoworms—70 nm in length and 30 nm in the mean diameter—accumulated in diseased tissues naturally or with the help of specific targeting ligands.^[^ ^99^ ^]^ The synthetic biomarkers (e.g., fluorophore‐labeled glutamate‐fibrinopeptide B) are cleaved off by the disease dependent proteases, and cleared into the urine where they are detected by mass spectrometry or ELISA (**Figure** **6**A).^[^ ^100^, ^101^, ^102^ ^]^ The urinary synthetic biomarkers, designed and optimized by Kwong and co‐workers, were able to noninvasively detect liver fibrosis, cancer,^[^ ^100^ ^]^ and thrombosis.^[^ ^101^ ^]^ To overcome the requirement of a mass spectrometer for sensing the urinary reporters, which precludes the global application of this method, Kwong and co‐workers redesigned their nanosensors to release a reporter that was functionalized with a distinct ligand for lateral flow assay (LFA) detection.^[^ ^102^ ^]^ The nanosensors were engineered by conjugating thrombin‐ or MMP‐9‐substrate reporter tandem peptides to PEG‐coated iron oxide nanoworms. When the linking peptides were cleaved off by the proteases (thrombin substrate: Pro‐Leu‐Gly‐Leu‐Arg‐Ser‐Trp and MMP substrate: Pro‐Leu‐Gly‐Val‐Arg‐Gly‐Lys), the reporter were cleared into the urine and captured onto a paper test stripe via ligand binding antibodies, creating a dark line on the paper stripe. Using these paper LFAs, the team was able to detect thrombosis and colorectal cancer with diagnostic accuracies.^[^ ^102^ ^]^ They then combined the developed existing single molecular array (SiMoA) technology with the synthetic biomarker nanosensors, and noninvasively detect thrombosis at the microdose‐scale.^[^ ^102^ ^]^

However, it was not possible to remotely control these nanosensors for the visualization of protease activity in disease tissue, thus the release of reporter elements into the urine after unspecific proteolysis can lead to off‐target activation. To reduce off‐target activation, Kwong and co‐workers developed a photoactivated spatiotemporally responsive nanosensor that is protected by photolabile molecules but unveiled by ultraviolet light. The peptides of the photoactivatable sensor, consisting of a MMP‐cleavable peptide chain and a fluorescent activity reporter, were coupled to the IONP. Photolabile molecules, 1‐(4,5‐dimethoxy‐2‐nitrophenyl) diazoethane (DMNPE), were coupled directly to the free carboxylic acid side chains of the MMP‐cleavable peptides (Pro‐Leu‐Gly‐Leu‐Glu‐Glu‐Ala). DMNPE functions as a removable barrier that blocked protease activity through steric hindrance. The signal of the fluorescent activity reporter was quenched once assembled on the IONP. In presence of 365 nm light, DMNPE was selectively removed by photolysis, unveiling the MMP cleavage site (Figure 6B). For in vivo tests, the team replaced the fluorescein‐labeled reporters (activity reporter) with the previously described urinary reporters, which were accumulated in the urine after proteolysis and quantified by mass spectroscopy or ELISA.^[^ ^100^, ^101^, ^102^ ^]^ In vitro and in vivo experiments demonstrated that photoactivatable nanosensors were protected from proteases and able to measure protease activity in tumor microenvironment after activation.

In in vivo experiments, the sensors were intravenously injected into healthy and tumor mice. Urine was voided after one hour to remove all unspecifically cleaved probes. The tumor side was then exposed to light and a second urine probe was taken 30 min later. While unprotected sensors did not distinguish between healthy and tumor mice, a 2.1‐fold signal increase was detected when the sensors in tumor mice were exposed to light. A 2.6‐fold signal increase was observed when comparing light treated sensors in tumor mice with sensors without any light exposure in healthy mice (Figure 6B).^[^ ^50^ ^]^

#### 2.1.2 Binding‐Dependent Activation

In the previous sections we discussed nanosensors that are activated via enzymatic catalytic events, more precisely by protease cleavage. As mentioned, another approach for activation via target‐interaction is the binding‐dependent activation, which will be discussed in the following chapters.

##### Molecular Beacon

A common approach for binding‐dependent activation of nanosensors are molecular beacons. These become activated through a conformational change caused by hybridizing with intracellular targets (e.g., RNA, DNA, proteins). The first part of this section will cover the “classical” molecular beacon that unfold through binding to RNA, DNA, or proteins and thus are activated. The second part describes the “nonclassical” molecular beacon, including nanoflares that are activated through dehybridization of the nanoflare from the probe.

- “Classical” Molecular Beacon

**Figure 7:**
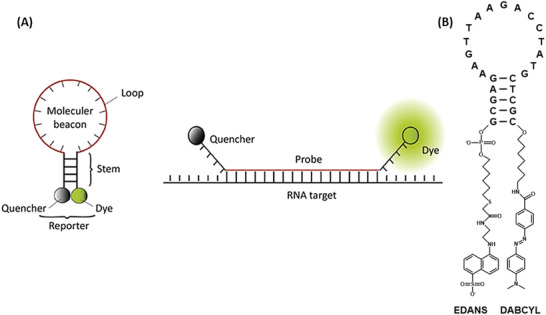
Working principal of “classical” molecular beacon. A) When inactive, “classical” molecular beacons consist of a hybridized stem region and a nonhybridized loop region (hairpin‐like structure). In the presence of the target structure, the stems regions dehybridize and the loop region hybridizes with target sequence, therefore, dye and quencher become separated. The sensor is now activated. Reproduced with permission.^[^ ^103^ ^]^ Copyright 2016, Elsevier. B) First molecular beacon. The oligonucleotide consisted of a 25‐nucleotid‐long sequence of which 5 nucleotides at each end formed the stem. Redrawn based on.^[^ ^104^ ^]^

“Classical” molecular beacons appear in a hairpin‐like structure composed of three parts: the reporter, usually containing a fluorophore at one end and a quencher on the other end, the stem, ensuring the hairpin structure, and the loop domain, usually consisting of 15–30 nucleotides partly complementary to the target sequence. In its native state, the fluorophore and quencher are in close proximity. Due to target hybridization, the hairpin structure opens up, separating quencher and fluorophore and restoring the fluorescent signal (**Figure** **7**A).^[^ ^103^ ^]^ The quenching effect of the molecular beacons relies on the same principle as for the protease‐based sensors previously described, i.e., energy resonance transfer of dye molecules in close proximity, FRET and SET.

The idea of molecular beacons goes even further back than the development of protease‐activated NIRF probes designed by Weissleder et al.^[^ ^9^ ^]^ The first molecular beacons were engineered by Tyagi et al. in 1996,^[^ ^104^ ^]^ consisting of the fluorophore 5‐(2ʹ‐aminethyl)aminonaphtalene‐1‐sulfonic acid (EDANS) and the quencher DABCYL, which were linked over a (CH_2_)_7_‐NH_2_ spacer in case of DABCYL and a (CH_2_)_6_‐SH spacer for EDANS to the stem. DABCYL was covalently linked to the 3ʹ hydroxyl group and EDANS to the 5ʹ phosphate group. Two oligonucleotide sequences were investigated: one with a 5 nucleotide long stem and a 15 nucleotide long loop, named probe A (Figure 7B) and another with a 8 nucleotide long stem and a 35 nucleotide long loop, termed probe B (nucleotide sequence probe A: 5ʹ‐GCGAGAAGTTAAGACCTATGCTCGC‐3ʹ and nucleotide sequence probe B: 5ʹ‐GCGAGTGCGCCTTAACTGTAGTACTGGTGAAATTGCTGCCATTGCACTCGC‐3ʹ, the underlined part marks the stem region). They noted that the most significant parameters affecting the conformational change of molecular beacons are the length of the stem and the loop region. Tyagi et al. observed that a stem sequence of 4–12 nucleotides formed a stable duplex, which is still short enough to dissociate during target hybridization. Further, the study revealed that the probe sequence should be at least twice as long as the arm region to ensure a conformational change. This probe only emitted a fluorescent signal when hybridizing to its target.^[^ ^104^ ^]^

To increase the quenching efficiency, especially in the NIR range, Dubertret et al. replaced DABCYL with a 1.4 nm diameter AuNP.^[^ ^15^ ^]^ Besides improving the quenching efficiency, DNA‐modified AuNPs were shown to offer couple advantageous properties, including the ability to enter cells, protect the DNA from enzymatic degradation and exhibit intracellular stability.^[^ ^105^ ^]^ Their study showed that hybrid materials, composed of a 25 nucleotide long single stranded DNA (ssDNA) covalently linked to an AuNP via a (CH_2_)_6_‐SH spacer and an organic dye (e.g., fluorescein, rhodamine 6G, Texas red or Cy5) via a (CH_2_)_7_‐NH_2_ spacer, served as molecular beacon with a quenching efficiency of 99.966 ± 0.026%. The molecular beacons were shown to detect single mismatches in random sequences.^[^ ^15^ ^]^

Several groups reported the ability of molecular beacons to detect and visualize intracellular mRNA,^[^ ^106^, ^107^, ^108^, ^109^ ^]^ which makes this technology a promising tool for diagnosis of diseases based on abnormalities in gene expression such as cancer. The first molecular beacon‐based methodology was reported by Peng et al.,^[^ ^17^ ^]^ which could detect cancer cells and monitor expression of tumor marker genes in viable cells. His laboratory prepared probes that either targeted survivin or cyclin D1 mRNA. The molecular beacons contained DABCYL as the quencher, and Texas Red (for visualization of cyclin D1), Cy3 or FITC (for visualization of survivin) as dye. The nucleotide sequence for survivin was as followed FITC‐5ʹ‐TGGTCCTTGAGAAAGGGCGACCA‐3ʹ‐DABCYL or Cy5‐5ʹ‐CTGAGAAAGGGCTGCCAGTCTCAG‐3ʹ‐DABCYL (the stem region is underlined). The molecular beacon for cyclin D1 detection had the following nucleotide sequence: Texas‐Red‐5ʹ‐TGGAGTTGTCGGTGTAGACTCCA‐3ʹ‐DABCYL. Survivin is one of the most tumor‐specific molecules involved in apoptosis inhibition, tumor associated angiogenesis and resistance to anticancer therapies.^[^ ^110^ ^]^ Cyclin D1 is shown to be overexpressed in breast cancer tissues, while is low or absent in normal tissues.^[^ ^111^ ^]^ Cyclin D1 plays a role in controlling the cell cycle, and its deregulation affects cellular process with potential oncogenic consequences, including angiogenesis, centrosome duplication and DNA damage response.^[^ ^112^ ^]^ Peng et al.^[^ ^17^ ^]^ reported the simultaneous detection of survivin and cyclin D1 with fluorescent intensity correlating with the level of gene expression using survivin and cyclin D1 molecular beacon concurrently. Strong fluorescent signals were detected for breast cancer cells, but not in normal breast cells, indicating the potential of this probe not only for identifying the level of expression of a variety of tumor marker genes but also for screening cancer drugs through the measurement of expression of multiple genes that are critical for drug response.^[^ ^17^ ^]^

The idea of simultaneous detection of multiple tumor mRNAs was adopted by several groups, because cancer is associated with a variety of mRNAs. Targeting only one tumor mRNA might lead to false positive as well as false negative results. Therefore these groups followed the similar idea for simultaneous detection of multiple tumor mRNAs by preparing multicolor nanoprobes.^[^ ^16^, ^105^, ^113^ ^]^

**Figure 8:**
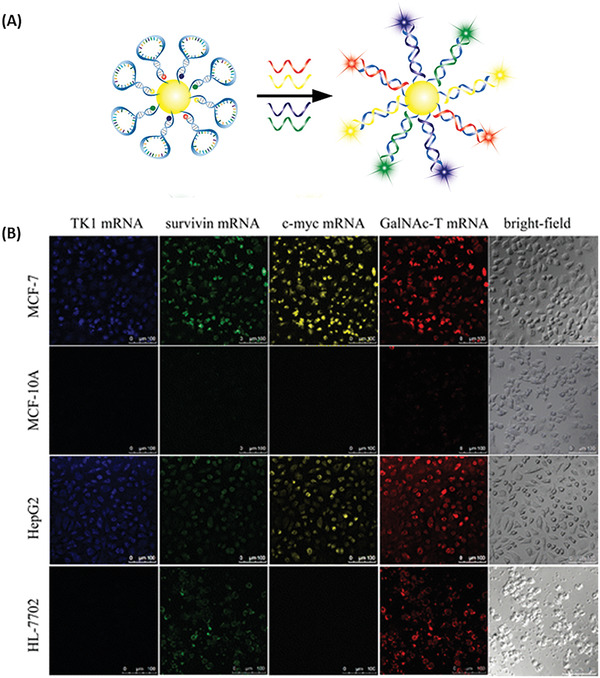
Multicolor nanoprobe. A) Activation of the four‐color nanoprobe through target hybridization, enabling multiplex detection of mRNAs, namely, TK1, survivin, c‐myc, or GalNac mRNA. B) Experimental results of the application of the four‐color nanoprobe in MCF‐7, MCF‐10A, Hep‐G2, and HL‐7702 cells. The cells were incubated with 1 × 10^−9^ m of four‐color nanoprobes for 3 h at 37 °C. Reproduced with permission.^[^ ^16^ ^]^ Copyright 2013, American Chemical Society.

Qiao et al.^[^ ^105^ ^]^ engineered an AuNP/bimolecular beacon (AuNP/bi‐MB) consisting of a 20 nm AuNP conjugated to FITC labelled survivin targeting molecular beacon (FITC‐5ʹ‐AGACAGTTGAGAAAGGGCTGCTGTCTAAAAAA‐3ʹ‐(CH_2_)_3_‐SH) and Cy5 labeled cyclin D1 targeting molecular beacon (Cy5‐5ʹ‐AGCTCAGAGTTGTCGGTGTAGATTGAGCTAAAAAA‐3ʹ‐(CH_2_)_3_‐SH) (**Figure** **8**A). The AuNP/bi‐MB was able to detect induced changes in gene expression in breast cancer cells, thus providing an efficient approach to reduce false positive and false negative results.^[^ ^105^ ^]^ The specificity of multiple detection with molecular beacons was further improved by Pan et al.^[^ ^16^ ^]^ They designed a four‐color nanoprobe that was able to simultaneously detect TK1 mRNA (associated with cell division and associated with tumor growth^[^ ^113^ ^]^), survivin mRNA, c‐myc mRNA (activator of tumorigenesis^[^ ^113^ ^]^) and GalNac‐T mRNA (expressing a key enzyme for the biosynthetic pathway of GM2/GD2, which are highly expressed on a variety of cancer cells^[^ ^113^ ^]^) in living cells (Figure 8A). The four‐color nanosensor was composed of four different molecular beacons that were linked to an AuNP. The molecular beacon were designed as followed: Alexa Fluor 405‐5ʹ‐ACGACGCCAGGGAGAACAGAAACCGTCGTAAAAAA‐3ʹ‐(CH_2_)_3_‐SH (TK1 mRNA), Alexa Fluor 488‐5ʹ‐GACATGTAGAGATGCGGTGGTCCATGTCAAAAAA‐3ʹ‐(CH_2_)_3_‐SH (survivin mRNA), Cy3‐5ʹ‐CAGTTGGTGAAGCTAACGTTGAGCAACTGAAAAAA‐3ʹ‐(CH_2_)_3_‐SH‐3ʹc‐myc mRNA), Cy5‐5ʹ‐CAGTGTCTTATGCGGATAGTGAAACACTGAAAAAA‐3ʹ‐(CH_2_)_3_‐SH (GalNAc‐T). In vitro experiments showed that the nanoprobe signal was dependent on the cell‐type. While the fluorescence of all four dyes was high in MCF‐7 cells, this was not the case for MCF‐10A cell, indicating an overexpression of TK1, survivin, c‐myc, and GalNAc‐T mRNA in MCF‐7 cells. In case of Hep‐G2 the fluorescence of all four dyes was high, indicating the overexpression of all four mRNAs. A low blue and yellow but a strong green and red fluorescence were detected in HL‐7702 cells, implying the overexpression of survivin and GalNAc‐T mRNA (Figure 8B). These results show that a multicolor nanoprobe can reduce the number of false negative and false positive results.^[^ ^16^ ^]^

Applying bioinformatics screens to design the complementary targeting sequence can increase the selectivity and sensitivity of the molecular beacons. Deng et al.^[^ ^114^ ^]^ used Basic Local Alignment Search Tool (BLAST) to identify the target mRNA sequence with the lowest similarity to whole human and mouse genomic mRNA and showed that the specificity of their previously designed STAT5B (Signal transducer and activator of transcriptional 5b) targeting molecular beacon was increased. STAT5B is a transcription factor involved in the proliferation and survival signaling in solid tumors such as breast cancer and prostate cancer.^[^ ^114^, ^115^ ^]^

**Figure 9:**
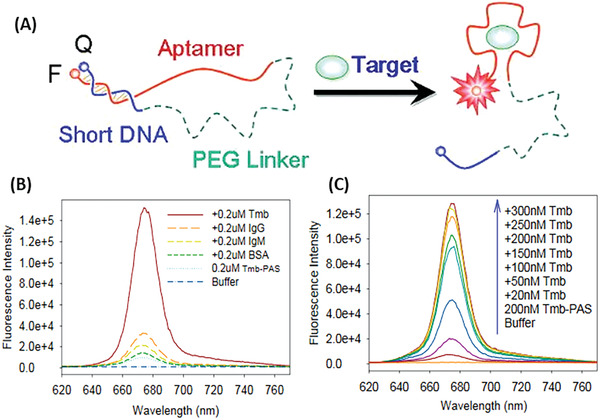
Protein targeting aptamer probe. A) Scheme of sensor activation. F is the abbreviation for fluorophore and Q for quencher. B) Fluorescence intensity dependence on the concentration of thrombin (Tmb), Immunoglobulin G (IgG), Immunoglobulin M (IgM) or Bovine serum albumin (BSA). C) Fluorescence intensity dependence on thrombin (Tmb) concentration. Reproduced with permission.^[^ ^116^ ^]^ Copyright 2008, American Chemical Society.

Instead of targeting mRNAs, Tang and colleagues engineered protein targeting aptamer probes that can detect adenosine triphosphate (ATP) and human‐*α*‐thrombin in buffer with high specificity. The sensor was composed of four elements: an aptamer, a short DNA sequence partly complementary to the aptamer, a PEG linker, and a fluorophore/quencher pair (Chlorin e6/Blackhole Quencher 2) linked to the termini of the sensor (ATP‐sensor: Ce6‐CACCTGGGGGAGTATTGCGGAGGAAGGTT‐(CH_2_CH_2_O)_36_‐CCAGGTG‐BHQ2 and human‐ *α*‐thrombin sensor: Ce6‐CCAAC‐(CH_2_CH_2_O)_30_‐GGTTGGTGTGGTTGG‐BHQ2). In its inactive state, the short DNA was hybridized with the aptamer, bringing quencher and fluorophore in close proximity. In presence of the target, the interaction of the aptamer and the target disturbed the conformation, resulting in the recovery of the signal (**Figure** **9**A). The two designed sensors were highly specific to their target. The fluorescent signal of the ATP‐sensor did not change much after addition of guanosine triphosphate (GTP), uridine triphosphate (UTP) or cytidine triphosphate (CTP), but increased significantly after adding ATP. Similar results were obtained for the human‐*α*‐thrombin sensor. The fluorescent signal was not significantly influenced by adding immunoglobulin G (IgG), immunoglobulin M (IgM), or bovine serum albumin (BSA) (Figure 9B), however adding 300 × 10^−9^ m *α*‐thrombin to sensor solution increased the fluorescent signal by up to 17.6 times (Figure 9C).^[^ ^116^ ^]^ A similar aptamer molecular beacon, targeting the cell membrane protein tyrosine kinase‐7 (associated with several cancers), was proposed by Shi et al. The sensor was composed of a hairpin‐like single stranded oligonucleotide containing the sgc8 aptamer, a poly‐T sequence, a stem region (which is also complementary to parts of the aptamer region) and the fluorophore/quencher pair BHQ1/Cy5 (Cy5‐5ʹ‐CTAACCGTTTTTTTTTTTTTTTTTTATCTAACTG CTGCGCCGCCGGGAAAATACTGTACGGTTAGA‐3ʹ‐BHQ2). In the presence of the cell membrane protein tyrosine kinase 7, the aptamer bound to the protein, initiating the conformational change of the molecular beacon and leading to the separation of quencher and fluorophore. The aptamer molecular beacon was able to image CCRF‐CEM cancer cells in mice, indicating the potential of the sensors for diagnosis of disease.^[^ ^7^ ^]^

b. “Nonclassical” Molecular Beacon

In the following chapter we will discuss different interpretations of the design and operating principle of molecular beacons, which we therefore summarize as “nonclassical” molecular beacons. These include nanoflare probes and sensors that are activated through different events such as an elongation processes.

**Figure 10:**
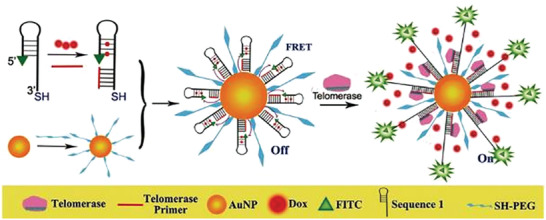
Synthesis and operating principle of the Dox containing‐gold nanoparticle (AuNP)‐molecular beacon. In the presence of telomerase activity, the telomerase primer was elongated, separating the dye (FITC) and the AuNP, thus restoring the signal and releasing the anticancer drug, Dox. Reproduced with permission.^[^ ^117^ ^]^ Copyright 2016, Wiley‐VCH.

*Activation through Elongation*: A recent study published by Ma et al.^[^ ^117^ ^]^ demonstrated the application of a molecular beacon that was able to monitor telomerase activity in living cells and deliver an anticancer drug to the diseased cells. Telomerase activity is associated with the uncontrolled division of cancer cells, making it a suitable target for the diagnosis and progression of cancer. The telomerase‐responsive sensor consisted of a PEG‐modified 15 nm AuNP that was functionalized with Doxorubicin (DOX)‐containing molecular beacons. The 5ʹ region of the molecular beacons showed the same sequence as the telomeric repeats and formed the stem. The 5ʹ ends were linked to FITC. The 3ʹ terminal ends were complementary to the telomerase primer (TP). The anticancer drug, DOX, was incorporated into the stem part of the probe. The basic design of the molecular beacon was as followed, but different numbers of telomeric repeats were investigated for optimization: FITC‐5ʹ‐AGGGTTAAAAAAAAAAAAAAAAAAAAAAACCCTAACTCTGCTCGACGGATT‐SH‐3ʹ. In case of telomerase activity, TP (sequence 5ʹ‐AATCCGTCGAGCAGAGTT‐3ʹ) bound to the 3ʹ terminal end and was elongated by the telomerase, causing the 5ʹ region to dehybridize and therefore restoring the fluorescent signal by separating FITC and AuNP. The dehybridization of the stem also causes the release of Dox (**Figure** **10**). This study successfully showed how molecular beacon can be used for diagnosis and also applied in a theranostic manner.^[^ ^117^ ^]^

**Figure 11:**
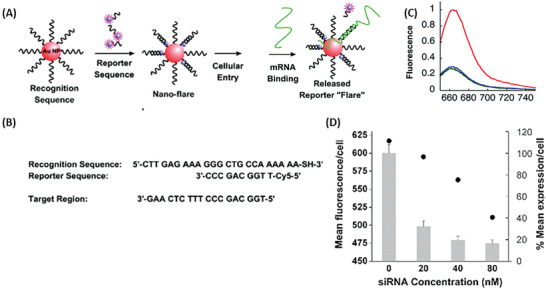
Operating principle and experimental results of nanoflare probes. A) Oligonucleotides serving as recognition sequence are conjugated onto gold nanoparticles (AuNP). When the complementary Cy5‐labeled reporter sequence (nanoflare) is added, it hybridizes with the AuNP and the fluorescence signal of Cy5 is quenched. The signal is restored, when the nanoflare dissociates from the recognition sequence because the target mRNA hybridizes to the recognition sequence and thus, displacing the nanoflare. B) Sequences of the recognition and reporter element and the target. C) Proof of concept of nanoflare sensor design. Fluorescence spectra of 1 × 10^−9^ m nanoflare sensors without the respective target (green), with the addition of 1 × 10^−6^ m target (red), and with the addition of 1 × 10^−6^ m of a noncomplementary target (blue). D) The graph shows the mean fluorescence per cell of the nanoflare (black dots) and the survivin expression per cell (gray bars) in dependence of the siRNA concentration. This indicates the influence of siRNA on nanoflare sensor activation and survivin expression. Reproduced with permission.^[^ ^119^ ^]^ Copyright 2007, American Chemical Society.

*Nanoflares*: A possible drawback of molecular beacons is an insufficient separation of the fluorophore from the quencher, because an efficient separation depends on the length of the oligonucleotide. A complete disconnection between quencher and fluorophore would be more effective.^[^ ^118^ ^]^ A complete separation of quencher and the fluorophores can be achieved with nanoflares. Nanoflares consist of a quencher particle functionalized with an oligonucleotide containing a specific recognition element for the target RNA^[^ ^113^, ^119^, ^120^, ^121^ ^]^ or molecules.^[^ ^122^ ^]^ In its inactive state, a fluorophore‐labeled short oligonucleotide, the so‐called nanoflare, is hybridized to the recognition element. Because of close proximity of fluorophore and quencher, no signal is detected. In presence of the target, the nanoflare is replaced, and the signal restored (**Figure** **11**). Seferos et al.^[^ ^119^ ^]^ developed a nanoflare for detecting survivin occurrence in breast cancer cells, using 13 nm AuNPs functionalized with 18‐base long recognition elements with each hybridized to a Cy5‐labeled nanoflare (Figure 11). The study showed that nanoflare probes had a lower background signal than “classical” molecular beacons, which was also due to a better nuclease resistance.^[^ ^119^ ^]^ Using an antisense oligonucleotide as the recognition element for targeting the mRNA region important for controlling the survivin expression the group was able to combine gene regulation and detection in a single system, making nanoflare probes a promising tool for theranostics.^[^ ^119^, ^120^ ^]^

To overcome the problem of false positive and false negative results, Prigodich et al.^[^ ^121^ ^]^ and Li et al.^[^ ^113^ ^]^ engineered multicolor nanoflare probes for detecting multiple tumor related mRNAs in living cells. Prigodich et al. designed a probe that was able to simultaneously sense survivin and actin (served as a control gene) in HeLa (high survivin expression) and Jurkat cell lines (low survivin expression) and differentiate between those two cell lines.^[^ ^121^ ^]^ The nanosensor developed by Li et al. was able to target three different mRNAs (c‐myc mRNA, TK1 mRNA, and GalNac‐T mRNA) and discriminate breast cancer and liver cancer cells from normal cells.^[^ ^113^ ^]^

##### Nanosensors Based on Graphene QD and Carbon Nanotubes

**Figure 12:**
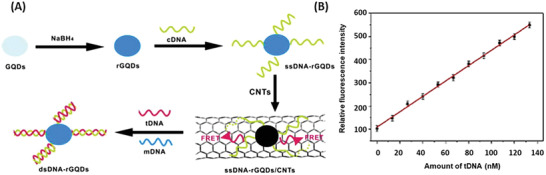
Operating principle and experimental data of ssDNA modified graphene quantum dots (GQD)—carbon nanotubes (CNT) based DNA sensors. A) In the presence of the target DNA (tDNA), the tDNA hybridizes with the single stranded DNA (ssDNA) on the GQD causing the probe activation (disabling FRET). B) Relative fluorescence intensity dependent on the amount of tDNA. The results indicate the sensors ability to measure tDNA concentrations with a linear range between 1.5 × 10^−9^ and 133.0 × 10^−9^ m and a detection limit of 0.4 × 10^−9^ m . GQD, graphene quantum dot; rGQD, reduced graphene quantum dot (reduction with NaBH_4_); cDNA, connecting DNA; CNT, carbon nanotubes; mDNA, single‐base mismatched ssDNA. Reproduced with permission.^[^ ^127^ ^]^ Copyright 2014, Elsevier.

Recently, graphene QD immunosensing has been demonstrated to detect human immunoglobulin G^[^ ^123^ ^]^ and cardiac Troponin I, a biomarker for myocardial infarction.^[^ ^124^ ^]^ Zhang et al. engineered a QD and carbon nanotube system for detecting target DNA with a selectivity down to a single mismatch.^[^ ^125^ ^]^ While metal ion‐based QDs often show high toxicity and blinking behavior, graphene QDs (GQD) have a low toxicity and stable emission. Furthermore carbon nanotubes are good electron acceptors in FRET, and show a strong *π*–*π* interaction (noncovalent forces, advantageous to covalent forces, because the latter can deform the carbon nanotubes^[^ ^126^ ^]^) between GQD and carbon nanotubes, making them an efficient FRET pair. Carbon nanotubes are also biodegradable and therefore suitable for in vivo application. For these reasons Qian et al.^[^ ^127^ ^]^ anticipated it would be advantageous to design a nanosensor based on GQD and carbon nanotubes.^[^ ^127^ ^]^ GQD functionalized with ssDNA (5ʹ‐NH2‐TTGGTGAAGCTAACGTTGAGG‐3ʹ) complementary to the target DNA (ssDNA‐GQD) were adsorbed on the carbon nanotubes via electrostatic and *π*–*π* interactions, thus linking the FRET donor and acceptor in close proximity and resulting in a quenched signal. The electrostatic and *π*–*π* interactions were disrupted in case of target DNA hybridization to the assembled ssDNA‐GQD carbon nanotubes, leading to the dissociation of dsDNA‐GQD from the carbon nanotubes. The separation of GQD and carbon nanotubes yielded in the recovery of the fluorescent signal (**Figure** **12**). Their results showed that the addition of target DNA recovered 85% of the signal. However single base mismatch ssDNA also led to a 50% recovery of signal intensity, but for every further mismatch the fluorescence recovery was very weak. Qian et al.’s approach was the first one to sense DNA via GQD and carbon nanotube interaction.^[^ ^127^ ^]^

### 2.2 Activation via Physiological Changes

The previous described sensors were activated via target interaction. The following chapter addresses sensor activation through changes in the physiological environment, e.g., the pH or ROS.

#### 2.2.1 pH dependent Activation

**Figure 13:**
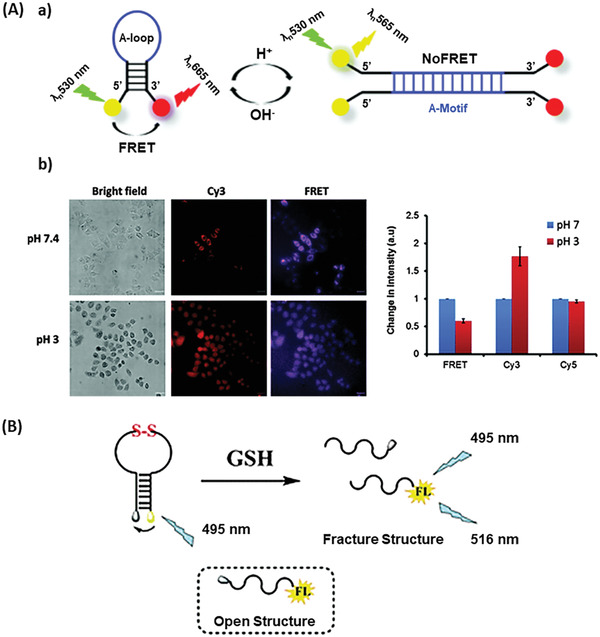
pH‐ and GSH‐triggered nanoprobes. A) Operating principle and experimental data of the pH sensing molecular beacon. a) Protonation of adenines causes the switch from a hairpin like structure to the A‐motif, disabling FRET. b) The pH sensing molecular cells were applied in HeLa cells. Live imaging was conducted with bright field and epifluorescence microscopy at pH 7.4 and pH 3. c) The diagram displays the change in intensity of FRET, Cy3 (donor), and Cy5 (acceptor) at a pH 7 and a pH 3. Reproduced with permission.^[^ ^18^ ^]^ Copyright 2016, Royal Society of Chemistry. B) GSH induces the separation of the two molecular beacon fragments via thiol‐disulfide exchange and consequently disabling FRET. Reproduced with permission.^[^ ^135^ ^]^ Copyright 2012, Royal Society of Chemistry.

Conformational changes, leading to probe activation, do not have to be initiated by a binding process. The conformation of DNA is often dependent on the protonation of the nucleobases, thus a varying pH may induce changes in the configuration of DNA.^[^ ^18^ ^]^ Based on the pH sensibility of DNA, several groups developed molecular beacon based DNA switches for pH sensing.^[^ ^18^, ^19^, ^20^, ^128^, ^129^, ^130^ ^]^ Many biological processes such as enzymatic catalysis, protein folding, cell proliferation and apoptosis rely on a specific intracellular pH.^[^ ^131^ ^]^ Therefore abnormal pH change is often associated with diseases such as cancer^[^ ^132^ ^]^ and Parkinson's and Alzheimer's.^[^ ^133^ ^]^ Narayanaswamy et al. successfully applied a pH depended nanosensor in living cells. Under normal physiological conditions (pH 7.4), the proposed sensor appeared in a hairpin‐like structure, consisting of 12 consecutive A‐bases in the loop region and a stem region containing 2 × 5 base pairs. The 5ʹ and 3ʹ end of the probe were labelled with Cy3 as FRET donor and Cy5 as FRET acceptor (Cy3‐5ʹGACGCCAAAAAAAAAAAACGCGTC‐3ʹ‐Cy5). The close proximity of the donor and acceptor dyes induced the FRET effect. A pH change toward more acidic conditions protonated the adenine nucleobases (AH^+^), causing the transformation of the closed hairpin structure into an open A‐motif through reverse Hoogsteen [AH^+^–H^+^A] hydrogen bonding interactions. The A‐motif was also stabilized by electrostatic interactions between AH^+^ and the phosphate backbone. The transformation into the A‐motif caused the separation of the donor and acceptor dyes, therefore inhibiting FRET (**Figure** **13**A). The sensor was successfully applied in live HeLA cells (Figure 13A).^[^ ^18^ ^]^ Besides the successful application of the A‐motif to measure the pH,^[^ ^18^ ^]^ other DNA structures, such as parallel (Hoogsteen) and antiparallel (Watson–Crick) strand duplexes^[^ ^20^ ^]^ and the I‐motif, were shown to be valuable and effective DNA structures for the pH measurement.^[^ ^128^, ^129^, ^130^ ^]^

A nonoptical imaging approach for pH sensing using Mn(II)‐containing layered double hydroxide (Mn‐LDH) nanoparticles was developed by Li et al.^[^ ^134^ ^]^ These sensors were shown to serve as a potential imaging agent for the diagnosis of cancer and were designed as an alternative to Gd(III)‐based T_1_‐weighted MRI contrast agents, because (Mn‐LDH) nanoparticles seem to offer a safer clinical approach.^[^ ^134^ ^]^ These contrast agents were shown to sensitively respond to the acidic environment (pH 5.0–7.0, i.e., the pH range in a tumor microenvironment) with excellent imaging performance and exhibited a longitudinal relaxivity at a lower pH (9.48 mm^−1^ s^−1^ at pH 5.0) compared to the relaxivity at pH 7.4 (1.16 mm^−1^ s^−1^), which may result from the Mn ion induced protonation of OH groups in Mn‐LDH.^[^ ^134^ ^]^

#### 2.2.2 Reducing Environment

Inspired by the idea of molecular beacons, Guo et al.^[^ ^135^ ^]^ reported the development of a disulfide‐bound molecular beacon (SSMB), which is activated in reducing environments. Like the previously described molecular beacon, the sensor consisted of a stem region that was covalently linked to a quencher at one end and to a fluorophore at the other end, and a loop region. In particular, the loop region contained a disulfide‐bond, enabling thiol‐disulfide exchange through glutathione (GSH), and leading to dissociation of the two fragments (FAM‐5ʹ‐GCTGGACAGAGTAT‐S‐S‐ATATCAATTTTTTTTAGTCCAGC‐3ʹ‐TAMRA, the underlined part indicates the stem region). The fracture structure induced a greater distance between the quencher and fluorophore, similar to the nanoflare probes, and therefore reducing the quenching compared to the “classical” molecular beacon (Figure 13B).^[^ ^135^ ^]^

#### 2.2.3 Reactive Oxygen/Nitrogen Species

**Figure 14:**
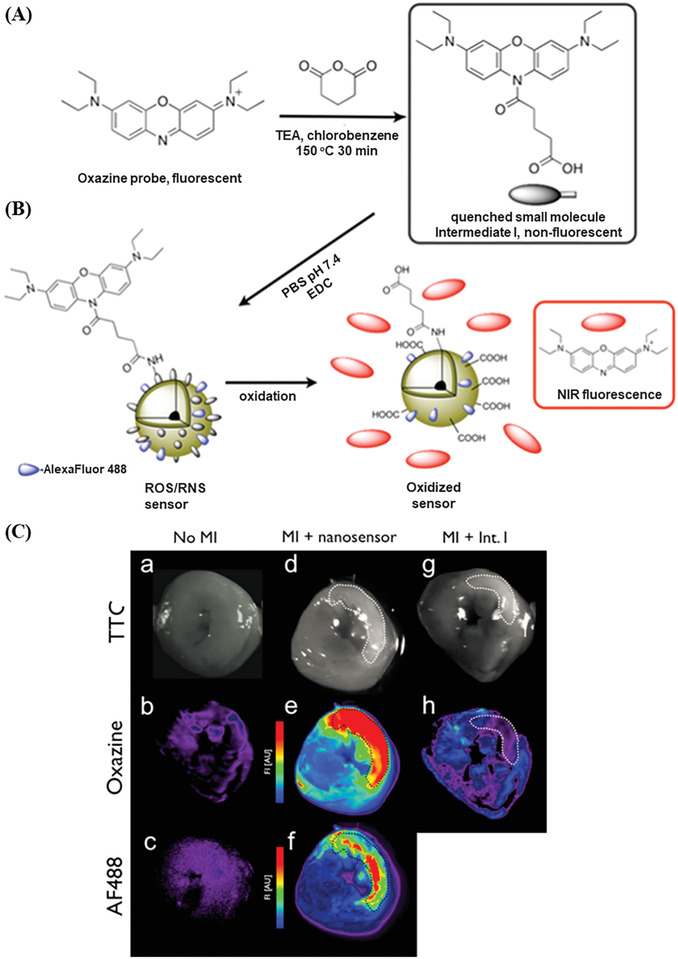
Synthesis, chemical structure, and experimental results of the ROS/RNS sensor. A,B) A quenched intermediate was generated with oxazine 1 and glutaric anhydride. The quenched intermediate was then linked to AlexaFlour488 labeled IONPs. In the presence of ROS, the oxazine dissociated from the IONP and became activated. C) Fluorescent reflectance imaging of coronal section of infarcted mouse heart. a–c) controls. 12–14 h after of myocardial infarction, d–f) the nanosensors or g,h) the intermediates were injected and the imaging was conducted 24 h later. Reproduced with permission.^[^ ^136^ ^]^ Copyright 2009, American Chemical Society.

Panizzi et al.^[^ ^136^ ^]^ synthesized and applied a reactive oxygen/nitrogen species (ROS/RNS) sensor that was activated by hypochlorous acid (HOCl/OCl^−^) generated from myeloperoxidase (MPO) and peroxynitrite (ONOO^−^), but stable toward oxidants such as hydroxyl radical, hydrogen peroxide and superoxide. ROS/RNS generated during inflammation is associated with the progress of a variety of diseases such as Alzheimer, atherosclerosis, cancer, etc., and MPO has been shown to be a biomarker of myocardial infarction and coronary artery disease. AlexaFlour488 labeled iron oxide nanoparticles with the average size of 41 nm were conjugated with oxazine fluorophore. While the oxazine fluorophore was quenched and only activated by ROS/RNS, AlexaFlour served as indicator of the probe's position (**Figure** **14**A,B). The ROS/RNS sensors showed favorable pharmacokinetics, localized in the myocardial infarction area in mice (Figure 14C) and the oxazine fluorescence was shown to coincide with the area rich in MPO, making the ROS/RNS nanosensor a promising technology to identify the sites of inflammation in atherosclerosis, cancer, metastasis and organ rejection.^[^ ^136^ ^]^

## 3 In Vitro Nanosensors

In many cases nanosensors designed for in vitro applications are similar to the in vivo ones and rely on the same or at least similar effects. In the following chapters we will first review in vitro nanosensors with similar working principles as the discussed in vivo sensors and then cover in vitro sensors with new principles such as field effect transistors, micro‐ and nanocantilevers and microfluidic purification chips.

### 3.1 Activation via Target Interaction

#### 3.1.1 Biocatalytic‐Dependent Activation

While the presented biocatalytic‐dependent in vivo nanosensors rely on protease activity, the spectrum of the here discussed biocatalytic‐dependent in vitro sensors is broader and covers the activation via protease, phosphatase, and kinase activities.

Several approaches relying on protease‐based activation and FRET inactivation have already been discussed previously, so it is no wonder that nanosensors relying on similar principles are also employed for in vitro arrays. Kim et al.^[^ ^137^ ^]^ engineered a nanosensor for monitoring the activity of proteases and their inhibition by measuring the energy transfer between AuNPs and QDs on a glass slide. Surface‐based assays limit aggregation of nanoparticles and can operate in a much smaller reaction volume, making it a more reliable, simple and sensitive approach compared to the solution‐based assay system, as previously designed by the same group.^[^ ^138^ ^]^ This assay exploited the strong interaction between biotin and streptavidin. Streptavidin labelled QDs were attached to a glass slide and interacted with AuNPs via avidin labelled peptides containing a recognition sequence for MMP‐7 (Cys‐Arg‐Pro‐Leu‐Ala‐Leu‐Trp‐Arg‐Ser‐Lys−biotin). FRET occurred as long as no proteases were added. Due to the successful detection of proteases and their inhibition, the proposed system could be used for diagnosing protease‐associated diseases and screening protease inhibitors.^[^ ^137^ ^]^

More interestingly, there are more enzymes suitable for probe activation such as phosphatases^[^ ^139^ ^]^ and kinases.^[^ ^139^, ^140^, ^141^ ^]^ An imbalance of phosphatase and kinase activity is associated with a variety of diseases. Phosphatases remove phosphate groups from proteins and therefore, play a pivotal role in signal transduction. Aberrant forms of phosphatases are shown to be involved in uncontrolled proliferation, differentiation, angiogenesis, and metastasis.^[^ ^142^ ^]^ Protein kinases modulate protein activity through phosphorylation of serine, threonine, or tyrosine side chains, and are also involved in signaling pathways. Their dysfunction is associated with diseases such as cancer.^[^ ^139^, ^141^ ^]^

**Figure 15:**
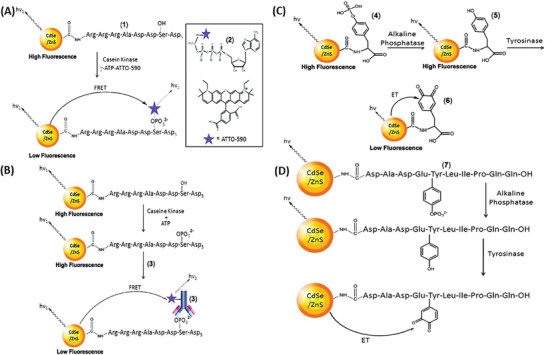
Chemical structure and sensor activation via protease and kinase activity. A,B) casein kinase 2 dependent activation. C,D) ALP dependent activation. Reproduced with permission.^[^ ^139^ ^]^ Copyright 2010, American Chemical Society.

Different approaches relying on kinase and phosphatase‐depended probe activation were presented by Freeman et al.^[^ ^139^ ^]^ The group designed two sensing probes for casein kinase (CK2) and alkaline phosphatase (ALP), respectively, as aberrant activity of CK2 is associated with Alzheimer, various types of cancer, and HIV‐1 transcription and ALP activity serves as an indicator for liver and bone diseases. Freeman et al.^[^ ^139^ ^]^ functionalized glutathione modified CdSe/ZnS QDs with peptides, which were recognized by CK2 and contained a serine unit for phosphorylation. Upon treatment with CK2 and *γ*‐adenosine triphosphate‐Atto‐590 (*γ*‐ATP‐Atto‐590), the fluorophore (Atto‐590) labeled *γ*‐phosphate was transferred to the serine unit, inducing FRET (**Figure** **15**A). In the second configuration, functionalized CdSe/ZnS QD reacted with ATP and CK2 and incubated with Atto‐590‐modified antiphosphoserine‐antibody, resulting in FRET as well (Figure 15B). To monitor ALP activity, the group designed *o*‐phospho‐l‐tyrosine‐modified CdSe/ZnS QDs, which were hydrolytically cleaved by ALP, yielding tyrosine‐modified CdSe/ZnS QDs. Through coaddition of tyrosinase, the tyrosine unit was oxidized to dopaquinone and quenched the luminescence of CdSe/ZnS QDs via electron transfer. The study showed that the quenching of the luminescence was enhanced as the ALP concentration increased (Figure 15C). In the second configuration, CdSe/ZnS QDs functionalized with phosphorylation peptides were treated with ALP and tyrosinase, resulting in hydrolysis, followed by oxidation, and yielding in a dopaquinone residue that induced the electron transfer (Figure 15D). While both CK2 detection methods showed a similar sensitivity, the first approach for monitoring ALP activity, in which the tyrosine residue was directly linked to the CdSe/ZnS QD (Figure 15C), resulted in a tenfold higher sensitivity than the second configuration (Figure 15D).^[^ ^139^ ^]^

The immunosensing strategy for kinase sensing was also applied by other groups to study the activity of human epidermal growth factor receptor 2 (Her2)^[^ ^140^ ^]^ and nonreceptor tyrosine kinase Abl and Scr (which play part in the progression of cancer).^[^ ^141^ ^]^ The sensing of Her2 activity was part of a multiplex detection assay for identifying the activity of urokinase type plasminogen activator (known to degrade the extracellular matrix and promote breast cancer invasion and metastasis) and Her2 (Her2 overexpression is associated with higher resistance to certain cancer treatments) simultaneously. The multiplex detection assay consisted of two sensors, i.e., one for kinase activity and one for protease activity. For the detection of urokinase‐type plasminogen (uPA), an uPA substrate was labelled with biotin at the N‐terminus (biotin‐Ser‐Gly‐Arg‐Ser‐Ala‐Asn‐Cys‐CONH_2_) and 1.4 nm AuNP at the C‐terminus. To determine the uPA concentration in a sample, the sample was treated with the substrate, followed by incubation with streptavidin functionalized QDs. Due to streptavidin–biotin interactions, the substrate was bound to the QDs. The intact substrate quenched the QD signals due to close proximity of AuNPs and QDs. The QD signal was shown when the substrate was cleaved. The human epidermal growth factor 2 (Her 2) peptide consisted of a terminal His tag, a spacer sequence, and a C‐terminal Her2 recognition sequence. In the presence of ATP, Her2 mediated phosphorylation of tyrosine residues. In the second step, the substrate was incubated with 3‐mercaptopropionic acid (MPA)‐capped QDs and dye‐labeled anti‐phosphotyrosine and the substrates bound to QDs via metal‐affinity coordination. In case of phosphorylated tyrosine residues, an immunocomplex was formed, inducing FRET.^[^ ^140^ ^]^

#### 3.1.2 Binding‐Dependent Interaction

The following chapters will discuss in vitro nanosensors that are activated via binding events which includes molecular beacons, sandwiching assays and single chip technologies.

##### Molecular Beacon

Several groups designed molecular beacon based arrays for detection of nucleic acids associated with various diseases such as West Nile, St Louis encephalitis,^[^ ^143^ ^]^ hepatitis B viruses,^[^ ^144^ ^]^ breast cancer,^[^ ^145^ ^]^ and genomic cystic fibrosis.^[^ ^146^ ^]^ Nanoflare probe based assays were reported to detect circulating tumor cells in whole blood samples,^[^ ^147^ ^]^ and interferon‐gamma (its expression is associated with various infectious diseases).^[^ ^148^ ^]^ Since the designs of molecular beacons and nanoflare sensors were already presented in the previous chapters, we will not go into detail with the just mentioned applications but introduce some further interpretations of molecular beacons.

**Figure 16:**
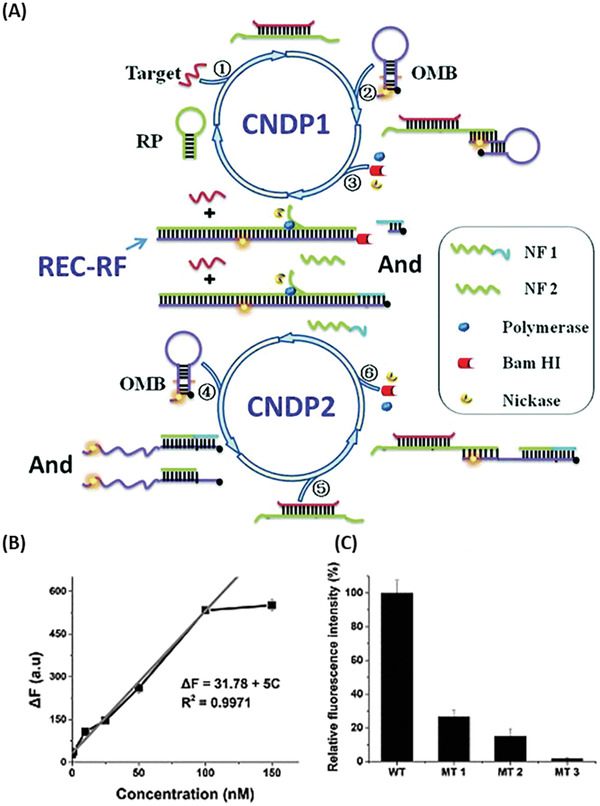
Operating principle and experimental results of the OMB‐based p53 sensing system. The process is structured into six steps: ① In presence of the target DNA, the recognition probe (RP) opens and hybridizes with its target; ② parts of the RP are not hybridized with the target and can hybridize with the overhang containing molecular beacon (OMB) creating a RP/OMB DNA hybrid; ③ the target DNA is released through the polymerization, followed by strand nicking in the presence of polymerase, nickase, and dNTPs. The release of the target causes the next initiation of strand‐displacement polymerization 1 (CNDP1). The left RP/OMB double strand is cleaved by BamHI, thus, quencher and fluorophore become separated and the two nicked fragments NF1 and NF2 have been released. ④ NF1 and NF2 initiate the second CNDP by hybridizing to OMB, ⑤ which then hybridize with the RPs. ⑥ The procedure of CNDP2 is similar to the one of CNDP1 and include the polymerization and nicking as well as the dissociation of the original strands. The new duplex is cleaved by BamHI. REC‐RF = restriction endonuclease cleavage‐restored fluorescence. B) Fluorescence dependence on p53 target DNA concentration in 5% calf serum. C) Relative fluorescence intensity of 100 × 10^−9^ m wild‐type and mismatch target DNA. The signal of the single‐mismatch DNA was at 26%, 15%, and 1.8% for MT1, MT2, and MT3, respectively. Reproduced with permission.^[^ ^118^ ^]^ Copyright 2016, Royal Society of Chemistry.

Le et al.^[^ ^118^ ^]^ proposed a sensor that relied on elongation processes and aimed to detect the tumor suppressor gene p53, whose mutation is associated with many human cancers. The sensor consisted of an overhang containing molecular beacon (OMB) and a recognition probe (RP). The latter is activated by the target DNA and served as a primer for OMB. Through cyclical nucleic acid strand‐displacement polymerization (CNDP) of OMB and cleavage through the restriction enzyme BamHI, the quencher and the dye molecule were separated from each other and the signal was restored (**Figure** **16**A). Even at a target concentration as low as 8.2 × 10^−12^ m, a significant fluorescent signal increase was detected through probe activation. A linear fluorescent signal increase was reported for a growing p53 target DNA concentration in 5% fetal calf serum (Figure 16B), indicating the probe's potential to even operate in a complex environment. Furthermore, the group showed that the fluorescence signal of the p53 sensing system was target DNA specific. Mismatch DNAs caused a strong decrease in fluorescence (Figure 16C).^[^ ^118^ ^]^

##### Sandwiching‐Based Sensor

**Figure 17:**
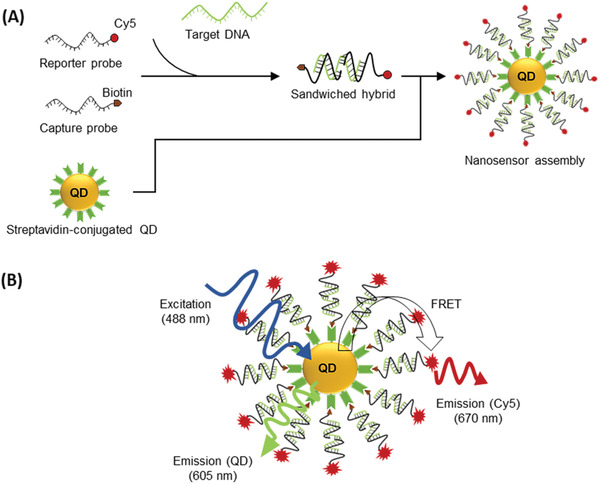
Nanosensors based on sandwiching the target nucleic acid. A) Reporter and capture probe hybridization to target DNA and gathering on the QD via biotin–streptavidin interaction. B) FRET between Cy5 and QD. Redrawn based on.^[^ ^149^ ^]^

Zhang et al.^[^ ^149^ ^]^ proposed a sensor that was activated via hybridization events. The hybridization did not cause conformational changes as in case of molecular beacon, instead the target DNA was sandwiched. The nanosensor was based on a streptavidin functionalized CdSe/ZnS QDs and two target‐specific oligonucleotides, one reporter oligonucleotide labelled with a fluorophore (Cy5 with different designs: Cy5‐5′‐GTTACCTTGACTAGC‐3′ or for Kras point mutation wild‐type: Cy5‐5′‐CTCTTGCCTACGCCAC‐3′ and mutant: Cy5‐5′‐CTCTTGCCTACGCCAA‐3’5) and a capture sequence labelled with biotin (5′‐TACGATAAGACAGAG‐3′‐biotin or in case of Kras point mutation: 5′‐CAGCTCCAACTACCA C‐3′‐biotin). In the presence of the target DNA (5′‐CTCTGTCTTATCGTAGCTAGTCAAGGTAAC‐3′), both oligonucleotides hybridized with the DNA and bound to the QD via biotin–streptavidin interactions, enabling FRET and making it possible to discriminate match and mismatch targets according to the fluorophore emission (**Figure** **17**A,B). The nanosensor was shown to be more sensitive than its molecular beacon alternative to detect Kras point mutation in ovarian serous borderline tumor (SBTs) clinical samples using the oligonucleotide ligation assay.^[^ ^149^ ^]^ Instead of using biotin–streptavidin interaction, Wang et al. coupled DNA and QD over carbodiimide coupling chemistry to each other and demonstrated nanosensor's potential to detect hepatitis B virus DNA and single‐base mutants.^[^ ^150^ ^]^

**Figure 18:**
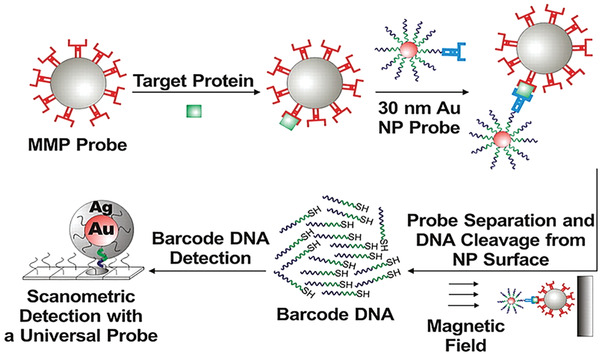
The AuNP‐IONP based bio barcode immunoassay. Design and operating principle of the AuNP‐IONP based bio barcode immunoassay. Reproduced with permission.^[^ ^154^ ^]^ Copyright 2009, National Academy of Sciences.

These approaches rely on a target DNA that is sandwiched by a reporter and capture probe. Nam et al.^[^ ^151^ ^]^ designed a bio‐barcode assay that relied on sandwiching a target antigen with monoclonal and polyclonal antibodies. The nanoparticle bio‐barcode assay detected prostate‐specific antigen (marker for breast and prostate cancer) at the low attomolar concentration. The nanoparticle‐based bio‐barcode assay relied on immunopolymerase chain reaction (PCR) but is faster and more sensitive. This is a significant factor because the concentration of prostate‐specific antigen (PSA) in breast cancer serum is much lower to that of normal men, making an ultrasensitive detection approach necessary. The nanoparticle bio‐barcode assay used two different probes, i.e., a PSA monoclonal antibody labelled magnetic microparticle (1 µm polyamine particle with iron oxide core) and an AuNP functionalized with hybridized oligonucleotides and polyclonal detection antibodies to target PSA. The magnetic and gold particles sandwiched the PSA target in solution. Applying a magnetic field, separated the particles and the bound AuNP from the rest of the sample. The barcode DNA was dehybridized and separated from the reacted particles using a magnet (**Figure** **18**). PSA was detected at 30 attomolarity and 3 attomolarity when applying a PCR.^[^ ^151^ ^]^ Further studies improved the bio‐barcode technology^[^ ^152^, ^153^ ^]^ and showed that the bio‐barcode assay was able to detect changes in the PSA level in clinical pilot trials earlier than commercially available assays.^[^ ^154^ ^]^ Other bio‐barcode assays were shown to detect markers such as interleukin‐2 (IL‐2) (IL‐2 is a human cytokine protein involved in inflammation and immune response and an indicator for infections through foreign antigens)^[^ ^155^ ^]^ and amyloid‐*β*‐derived diffusible ligands (ADDLs) in cerebrospinal fluid (CSF) of individuals (ADDLs is an indicator for Alzheimer).^[^ ^155^ ^]^

##### Multiplex Detection via Single Chip Technology

**Figure 19:**
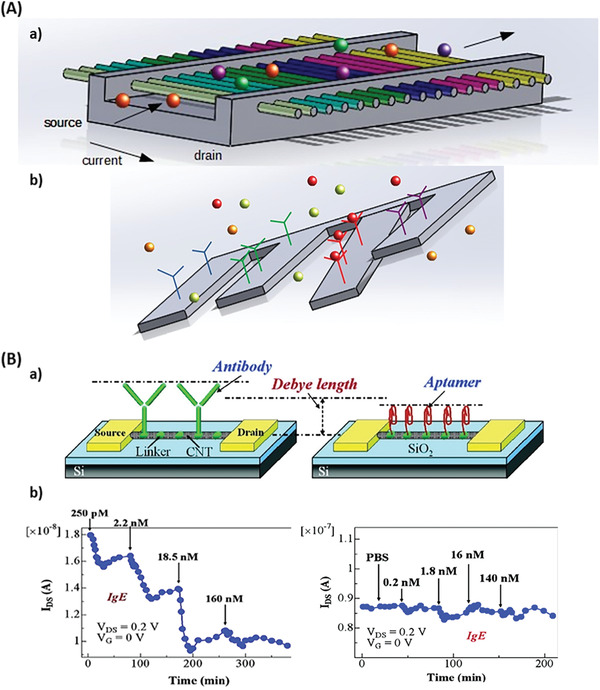
FET and cantilever based assays. A) Schema of nanowire based FET and cantilever assay. The colored circles represent different molecules. a) Schema of a nanowire‐based FET assay. When the molecules bind to the functionalized nanowires, the voltage is changed, thus, the conductance is altered. b) Schema of a nanocantilever. The biomolecules bind to the respective antibodies causing the cantilever to bend. The bending is then detected, e.g., with a laser or electronically. Redrawn based on^[^ ^156^ ^]^ B) Schema and experimental data of antibody or aptamer functionalized carbon nanotube FETs (CNT‐FETS). a) Schema of antibody or aptamer functionalized carbon nanotube FETs (CNT‐FETS). c) Source–drain current in dependence of the time and the addition of different concentrations of the target molecule (IgE). The left graph shows the source–drain current of an IgE‐aptamer functionalized CNT‐FET, while the right graph shows the source–drain current of a CNT‐FET functionalized with IgE‐monoclonal antibodies. Reproduced with permission.^[^ ^158^ ^]^ Copyright 2007, American Chemical Society.

Field effect transistor (FETs) and nanocantilever arrays (**Figure** **19**A) are reported to enable high multiplex detection of an entire proteome. These approaches are expected to offer reliability and a high low‐cost production.^[^ ^156^ ^]^ Therefore, the following chapters covers single chip technologies.

- Field Effect Transistors

Carbon nanotubes (CNT) and nanowires (NW) have been used in FETs.^[^ ^156^, ^157^, ^158^, ^159^, ^160^ ^]^ In a usual FET, the conductance is depended on the gate voltage. The gate voltage dependence of a FET is achieved by using n‐ or p‐doped semiconductors. Depending on the voltage, the free charge carrier can create a current channel with a voltage dependent diameter. The adsorption or binding of charged molecules is analogous to a gate voltage change, thus changing conductivity (Figure 19A).^[^ ^156^, ^157^, ^158^, ^159^ ^]^

CNT and NW can be functionalized with DNA, RNA or antibodies (immunological field effect transistors (immunoFET)) for detection. However, one major drawback of immunoFETs is the excess of the Debye length by the antibody.^[^ ^158^, ^161^ ^]^ Aptamers are usually smaller than the typical Debye length,^[^ ^161^ ^]^ consequently FETs functionalized with aptamers were reported to perform better than immunoFETs under similar conditions (Figure 19B).^[^ ^158^ ^]^ To name a few applications of aptamer modified CNT‐FETS, thrombin‐ and IgE‐aptamer functionalized CNT‐FETS were shown to successfully detect their target with a detection limit at 10 × 10^−9^ and 250 × 10^−12^ m, respectively.^[^ ^157^, ^158^ ^]^ The cancer biomarker cathepsin E was detected with concentrations low as 10 ng/ml in diluted human serum using cathepsin E binding aptamer functionalized CNT‐FETs.^[^ ^160^ ^]^ However, maintaining the semiconductor property of CNT during growth and surface modification was very difficult, making NW a promising alternative.^[^ ^159^, ^161^ ^]^ The application of NW is versatile^[^ ^162^ ^]^ and label‐free NW have been reported to detect nucleic acids,^[^ ^163^ ^]^ neurotransmitter (e.g., *γ*‐aminobutyric acid, a Parkinson's disease, and Meningitis associated neurotransmitter),^[^ ^164^ ^]^ various cancer biomarkers,^[^ ^161^, ^165^, ^166^, ^167^ ^]^ and viruses^[^ ^168^ ^]^ in real time with high sensitivity. Even though these sensing systems appear in the chapter for in vitro nanosensors, FETs offer the advantage of miniaturization. Therefore, they have the potential to be additionally applied in vivo as implantable biochips for the *γ*‐Aminobutyric acid sensing NW‐FET.^[^ ^164^ ^]^

b. Micro‐ and Nanocantilever Arrays

Micro‐ and nanocantilever arrays are micromechanical systems, which are typically microfabricated in silicon or a piezoelectrical material such as quartz and can be pictured as functionalized miniature diving boards anchored to a large mass, operated either statically or dynamically (Figure 19A).^[^ ^7^, ^156^, ^169^ ^]^ The two surfaces of the cantilever are made up of different materials (e.g., gold and silicon). When molecules adsorb at one side, a different surface stress is induced on each side, causing the cantilever to bend. This is known as the static mode and can be recorded as the beam reflection. Making use of this working principle, several groups demonstrated the potential of cantilever‐based optical deflection assays to detect DNA mismatches and study protein interactions (e.g., A‐immunoglobulin and PSA).^[^ ^170^, ^171^ ^]^

The dynamic approach relies on mass changes induced by nucleic acid hybridization or biomolecule–ligand interaction, which can be measured through the shift of resonance frequency. Resonating cantilevers were successfully applied for the detection of DNA,^[^ ^172^ ^]^ biomolecules such as PSA,^[^ ^173^, ^174^, ^175^ ^]^ and C‐reactive protein.^[^ ^176^ ^]^ Therefore, cantilevers are a promising tool for cost‐efficient, label‐free, and simple multiple biomolecular detection with high throughput.

c. Microfluidic Chips

Microfluidic chip is a promising and fast developing tool for POC diagnostics and versatile designs and applications have been introduced by many groups..^[^ ^177^, ^178^, ^179^, ^180^, ^181^ ^]^ Nanosensors (including CNT, NW, and cantilevers) offer label‐free biomarker detection with high throughput. However, the clinical relevance of these nanosensors is often limited by the requirement of a purified buffer because the direct application of physiological fluids can cause biofouling, nonspecific binding, or falsified signals. Therefore, Stern et al.^[^ ^180^ ^]^ designed a microfluidic purification chip (MPC) that captured biomarkers from physiological fluids, transferred these to a purified buffer to pass through a silicon nanoribbon detector. The avidin‐functionalized chip was treated with antibodies labelled with biotinylated and photocleavable DNA. When the blood sample was loaded into the chip, the biomarkers were captured and the chamber washed, and then perfused with the sensing buffer. Through irradiation with UV light, the photolabile group was cleaved, releasing the antibody–antigen complex into the buffer to react with the nanosensor. Using whole blood samples spiked with two cancer antigens, PSA, and carbohydrate antigen 15.3 (CA15.3), Stern et al.^[^ ^180^ ^]^ demonstrated biomarker detection by MPCs from physiological fluids. Furthermore MPCs lowered the need for ultrasensitive electronic detection because biomarkers were preconcentrated in the sample.^[^ ^180^ ^]^

The McDevitt laboratory proposed different approaches for a programmable Bio Nano Chip (pBNC) for the detection of disease biomarkers, such as the ovarian cancer associated cancer antigen 125 (CA125)^[^ ^177^, ^178^ ^]^ and HE4, MMP‐7 and CA72‐4.^[^ ^178^ ^]^ The “programmable” refers to the ability of the chip to be redesigned for different biomarkers. As proof of concept, the group designed CA125 sensing pBNC. The sample analysis was based on antibody modified agarose microbeads. The antibodies were biomarker specific. The presence of the biomarker induced immunofluorescent‐sandwiching and thus, enabled the detection.^[^ ^177^ ^]^ To improve early detection and the limitation of false negative results due to a low CA125 expression level in some cancers, the group optimized the chip design for multiplex detection of the following ovarian cancer associated biomarkers: CA125, HE4, MMP‐7, and CA72‐4.^[^ ^178^ ^]^ The microfluidic chip detection approach was further improved by coupling it to machine‐learning algorithm. The biomarker concentration results were transferred to the cardiovascular disease‐trained machine learning system that created a cardiac score indicting the cardiac health.^[^ ^182^ ^]^ Technically speaking the detection systems designed by the McDevitt laboratory did not employ nanosensors but the systems indicate the potential of microfluidic chips in diagnostics and the idea of introducing nanosensors in a programmable, learning microfluidic chip technology for multiplex detection and integrated interpretation.

d. Diagnostic Magnetic Resonance System

A biosensor platform relying on magnetic nano‐ and microparticles was designed by Lee et al.^[^ ^183^ ^]^ The proposed miniaturized diagnostic magnetic resonance (DMR) system based on the same principle was designed by Zhao et al. as the bi‐biotinylated peptide‐magnetic resonance switcher (BBP‐MRS).^[^ ^12^ ^]^ In the presence of substrate, magnetic nanoparticles changed to a clustered state, leading to an increase in *T*_2_ relaxivity. However, in this case the clustering was based on the affinity of ligands conjugated to the particles, which bound to the molecular target and caused a decrease of spin–spin relaxation time. The DMR system consisted of four major components, i.e., planar microcoils, microfluidic network, on‐board NMR electronics, and a permanent magnet. Lee et al.^[^ ^183^ ^]^ demonstrated the potential of the chip‐NMR nanosensor to detect infectious agents (using *Staphylococcus aureus*), mammalian cells (using mouse macrophages), and multiple protein biomarkers (e.g., epidermal growth factor receptor (EGFR) and HER2/neu).^[^ ^183^ ^]^

### 3.2 Activation via Physiological Changes

**Figure 20:**
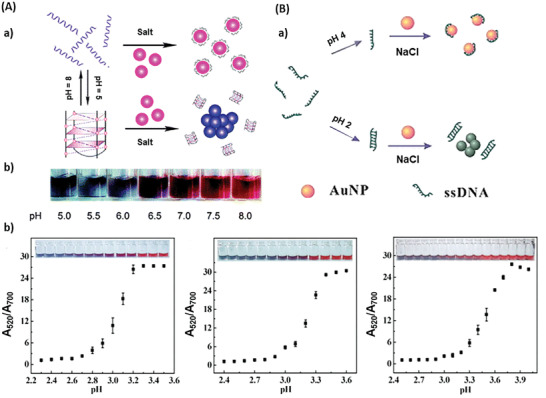
pH sensing nanosensors. A) I‐Motif DNA/AuNP based nanosensor. a) Operating principle of the sensor. At low pH (pH 5), the sensor appears in a quadruplex structure, thus, the AuNPs are free to aggregate. At a higher pH (pH 8), the ssDNA is free to interact with the AuNP, prohibiting AuNP aggregation. b) Colorimetric change due to interaction of I‐Motif DNA with AuNP. Reproduced with permission.^[^ ^184^ ^]^ Copyright 2008, Royal Society of Chemistry. B) homopolymeric deoxyadenosines/AuNP based nanosensor. a) Schema of working principle. b) Influence of different concentration of homopolymeric deoxyadenosines with 15 adenosines (left 25 × 10^−6^ m, middle 20 × 10^−6^ m, right 14 × 10^−6^ m) on the pH threshold (pH 3.2 for 25 × 10^−6^ m, 3.4 for 20 × 10^−6^ m and 3.7 for 14 × 10^−6^ m). Reproduced with permission.^[^ ^185^ ^]^ Copyright 2012, Elsevier.

Measuring the level of pH using the colorimetric approach is highly attractive since pH is a critical indicator of numerous diseases related to biological processes. Thus, nanosensors for measuring the pH in a medium have been designed by some groups.^[^ ^184^, ^185^ ^]^ Some of these pH sensors were based on the principle that unmodified AuNPs aggregated upon addition of salt but were dispersed in combination with ssDNA. The transition of AuNPs from a dispersed structure into aggregates is accompanied by a color change from red to blue. Applying this principle and using pH dependent DNA structures, such as cytosine rich ssDNA (e.g., CCCTAACCCTAACCCTAACCC, which appears in form of an I‐motif at a low pH and transforms into ssDNA at a high pH) (**Figure** **20**A) and poly deoxyadenosine (dA) ssDNA (e.g., 5′‐AAAAAAAAAAAAAAA‐3′, 5′‐AAAAAAAAA A‐3′, etc., that appear in single helices at the neutral pH and transforms into parallel duplexes at a lower pH) (Figure 20B), Chen et al.^[^ ^184^ ^]^ and Zeng et al.^[^ ^185^ ^]^ prepared pH dependent sensors for in vitro applications.^[^ ^184^, ^185^ ^]^ In addition to pH sensors based on AuNPs and DNA structures, colorimetric pH sensors based on polymers^[^ ^186^ ^]^ or AuNPs modified with protein antigens^[^ ^187^ ^]^ were also reported. An ionic liquid nanosensor for colorimetric pH detection based on phosphonium salt was reported by Das et al.^[^ ^188^ ^]^ This fluorescent salt was prepared by combining trihexyltetradecylphosphonium cation [TTP]^+^ with monoanionic [FL]^−^ and dianionic [FL]^2−^ fluorescein, yielding contrast colorimetric responses in acidic and neutral conditions.^[^ ^188^ ^]^

## 4 Concluding Remarks and Perspectives

Nanosensors offer great potential for diagnosis of diseases. The progresses made in the last two decades are remarkable and a variety of obstacles have already been overcome. However, there are still many difficulties that preclude clinical applications. Engineering in vivo nanosensors brings along the struggle of biocompatibility, cytotoxicity, and nuclease resistance. A low signal‐to‐noise ratio, blinking behavior, and false positive or false negative results impair a probe's reliability and sensitivity. Even if these drawbacks are overcome, high production costs limit the sensor application.

A lot of nanosensors presented in this review rely on optical detection based on FRET. The success of using FRET is dependent on the quenching efficiency and a good separation of the FRET pair. However, the separation of the FRET donor and acceptor in “classical” molecular beacons is often insufficient because the separation efficiency is dependent on the oligonucleotide length. Designing longer oligonucleotides is problematic, since the stem length needs to be short enough to dehybridize during target hybridization and the increase of the loop length reduces the specificity.^[^ ^103^ ^]^ Several groups addressed this problem of insufficient separation of the FRET pair by engineering nanoflare probes,^[^ ^113^, ^119^, ^120^, ^121^ ^]^ overhang molecular beacons,^[^ ^118^ ^]^ or target DNA sandwich assays.^[^ ^149^, ^150^ ^]^

Another problem of using fluorescent molecules for optical detection is that the fluorescence intensity does not necessarily correlate with the quantity of the target, because the fluorescent signal depends on the position of the lesion. Dual fluorochrome probes have been shown to be a promising solution to overcome this obstacle. These probes consist of an activatable sensor and a second nonactivatable “always‐on” probe. The latter serves as internal standard or reference probe. The ratio of the two signals is independent to the position of the lesion.^[^ ^33^, ^81^ ^]^

The major drawbacks of nucleic acid based nanosensors are the lack of stability and nuclease resistance in biofluids. For these reasons, several groups proposed hybrid materials composed of nucleic acid conjugated to nanomaterials such as AuNP or IONP. These hybrid materials have been shown to reduce enzymatic degradation and improve the intracellular stability of nucleic acids, the quenching effect on fluorescent dyes and the ability to enter cells.^[^ ^105^, ^145^ ^]^ However they are bigger in size, which also limits their application. Another strategy is to incorporate nuclease resistant building blocks (e.g., locked nucleic acids) into the nucleic acid based sensors.^[^ ^145^ ^]^ More research is still needed to optimize the sensor designs to achieve sensor stability and degradation resistance.

Sensor delivery into the cytosol, while the functionality is maintained, is another crucial point. Nanomaterials, such as IONP and AuNP, are often taken up into cells naturally depending on their size, shape, and surface chemistry.^[^ ^189^ ^]^ However, oligonucleotide‐based probes, e.g., molecular beacons, are often unable to pass the cell membrane.

Furthermore, more research should focus on multiplex biomarker detection because the detection of only one specific biomarker can lead to false positive or false negative results. Especially in the early stage of disease, biomarkers can present in inadequate quantities.

Cost‐effectiveness is a further aspect that needs to be considered. Nanosensors for disease diagnosis should offer high reliability, specificity, and sensitivity. Additionally, it is worthwhile to design sensors in a manner of simple application and low‐cost production, allowing easy clinical translation at a global scale.

## Conflict of Interest

The authors declare no conflict of interest.
